# A Regioselective Synthesis of Novel Functionalized Organochalcogen Compounds by Chalcogenocyclofunctionalization Reactions Based on Chalcogen Halides and Natural Products

**DOI:** 10.3390/molecules26123729

**Published:** 2021-06-18

**Authors:** Maxim V. Musalov, Vladimir A. Potapov, Vladimir A. Yakimov, Maria V. Musalova, Arkady A. Maylyan, Sergey V. Zinchenko, Svetlana V. Amosova

**Affiliations:** A. E. Favorsky Irkutsk Institute of Chemistry, Siberian Division of The Russian Academy of Sciences, 1 Favorsky Str., 664033 Irkutsk, Russia; musalov_maxim@irioch.irk.ru (M.V.M.); yakimov@irioch.irk.ru (V.A.Y.); musalova@irioch.irk.ru (M.V.M.); maylyan@irioch.irk.ru (A.A.M.); svz@irioch.irk.ru (S.V.Z.); amosova@irioch.irk.ru (S.V.A.)

**Keywords:** thymol, carvacrol, selenium dibromide, tellurium tetrachloride, tellurium tetrabromide, sulfur dichloride

## Abstract

The regioselective synthesis of novel functionalized condensed organochalcogen compounds by chalcogenocyclofunctionalization reactions based on chalcogen halides and the natural products thymol and carvacrol has been developed. The reactions of selenium dibromide with allyl thymol and allyl carvacrol proceeded in methylene chloride at room temperature in the presence of NaHCO_3_ affording bis[(7-isopropyl-4-methyl-2,3-dihydro-1-benzofuran-2-yl)methyl] and bis[(4-isopropyl-7-methyl-2,3-dihydro-1-benzofuran-2-yl)methyl] selenides in 90–92% yield. Similar sulfides were obtained in 70–72% yields by the reaction of sulfur dichloride in chloroform under reflux. Trihalotellanes containing the same organic moieties were synthesized from allyl thymol, allyl carvacrol and tellurium tetrachloride or tetrabromide in quantitative yields. Corresponding functionalized ditellurides were prepared in 91–92% yields by the reduction of the trichlorotellanes with sodium metabisulfite in two-phase solvent system. The comparison of reactivity of sulfur, selenium and tellurium halides in chalcogenocyclofunctionalization and distinguishing features of each reaction were discussed.

## 1. Introduction

Natural products and their derivatives play an important role in the discovery of new drugs [1,2,3,4,5]. Many modern drugs have been developed from natural products, and synthesis of novel functionalized compounds based on natural products is promising in terms of the possible manifestation of biological activity. 

The present work is devoted to regioselective synthesis of novel condensed functionalized organochalcogen compounds by chalcogenocyclofunctionalization reactions based on selenium dihalides, tellurium tetrahalides and sulfur dichloride and natural products, thymol (2-isopropyl-5-methylphenol) and carvacrol (5-isopropyl-2-methylphenol). Thymol and carvacrol are isomeriC-Natural monoterpenoid phenols, found in essential oils of *Thymus vulgaris* (thyme) and *Origanum vulgare* (oregano) and extracted from various other kinds of plants.

The thyme herb has been used in folk traditional medicine as a sedative and antiseptic since ancient times [6]. Ancient Egyptians used thyme for embalming. The ancient Greeks used it in their baths and burned it as incense in their temples, believing it was a source of courage. The spread of thyme throughout Europe was thought to be due to the Romans, as they used it to purify their rooms and to give an aromatic flavor to some food and liqueurs [7]. Its extract was used as a natural antibacterial gargle for sore throat and colds. 

Not only thymol and carvacrol derivatives, but these natural products themselves show various types of biological activity [8,9,10,11,12,13,14,15,16,17,18]. Thymol is widely used in the chemical industry and pharmacotherapy due to its antibiotic [9,10], anticancer [11], insecticidal [12,13] and antileishmanial [14] properties and general non-genotoxicity or cytotoxicity on human cells [15,16,17]. Besides, thymol is applied as an active antiseptic ingredient in some toothpastes, medicinal ointments and drugs for inhalation. This natural product is also proposed as an environmental-friendly, rapidly degrading and non-persisting pesticide [8]. 

**Figure 1:**
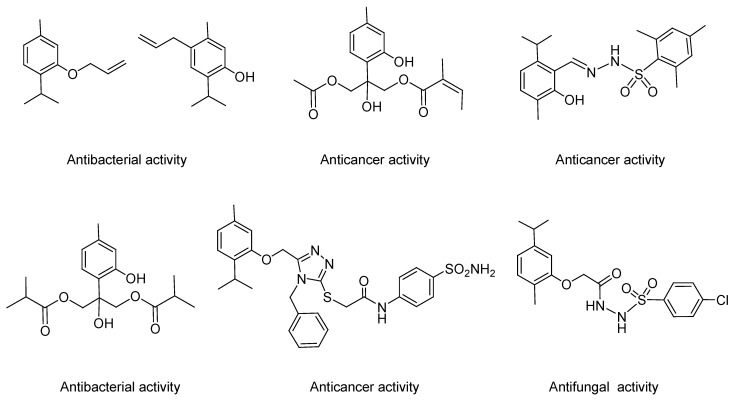
Known derivatives of thymol and carvacrol exhibiting antibacterial [18,22,23], anticancer [18,35,36,37] and antifungal [27] activities.

Derivatives of thymol and carvacrol exhibit a variety of biological activities [18,19,20,21,22,23,24,25,26,27,28,29,30,31,32,33,34,35,36,37] including antibacterial [18,22,23,24], antifungal [27,28,29], antitubercular [32] and anticancer [35,36,37] properties (some examples of biologically active thymol and carvacrol derivatives are presented in Figure 1). Simplest derivatives of these natural products such as allyl ether of thymol and allyl-substituted thymol (4-allyl-2-isopropyl-5-methylphenol) display antibacterial activity [18].

The discovery of the biological role of selenium gave a powerful impetus to the rapid development organoselenium chemistry, which is currently occupied an important place in chemical research. It is worth noting that organoselenium compounds and especially selenium-containing heterocycles show a variety of biological activities including antibacterial, antitumor, anti-inflammatory, neuroprotective and glutathione peroxidase-like actions [38,39,40,41,42,43,44,45,46,47].

In recent years, chalcogen-induced cyclization and especially selenocyclofunctionalization reactions have acquired particular importance as valuable tools for modern organic synthesis. The growing interest in selenocyclofunctionalization was demonstrated in excellent books and reviews [45,46,47,48,49,50,51,52,53,54]. 

Similar methodology of intramolecular halocyclization of alkenes containing nucleophilic functions which afford useful heterocyclic compounds including nitrogen heterocycles are well documented in the literature [55,56,57]. However, opportunities of selenocyclofunctionalization reactions have not yet been fully realized.

Addition products of selenenyl halides to alkenes are very reactive in nucleophilic substitution reactions since halogen atoms in β-haloorganyl selenides are activated by anchimeric assistance of the selenium atom [58]. The high anchimeric assistance effect of the selenium atom plays an important role in selenocyclofunctionalization reactions which include intramolecular nucleophilic substitution. This effect leads to considerable activation of halogen atoms to nucleophilic substitution and intramolecular cyclization reactions proceed very smoothly. It has been found that the anchimeric assistance effect of the selenium atom is approximately two orders of magnitude greater than the analogous effect of the sulfur and nitrogen atoms [58].

Previously, we first introduced selenium dihalides in organic synthesis for preparation of organoselenium compounds [59,60,61] and used these reagents for selenocyclofunctionalization reactions [62,63,64,65,66,67,68,69]. The application of selenium dihalides in selenocyclofunctionalization allows to carry out two heterocyclization processes in one molecule and to obtain selenides containing two heterocyclic moieties [62,63,64,65,66,67,68,69]. 

**Scheme 1:**
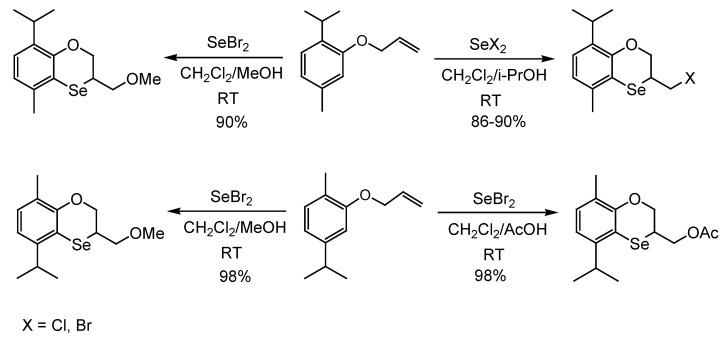
Synthesis of novel condensed products by the annulation reactions of selenium dibromide with allyl carvacryl and allyl thymyl ethers.

Previously we studied annulation reactions of selenium dihalides with allyl thymyl and allyl carvacryl ethers (Scheme 1) [70,71]. A novel methodology to accelerate annulation reactions leading to condensed selenium heterocycles was developed. The reactions of selenium dihalides with allyl thymyl and allyl carvacryl ethers (as well as with methyleugenol, allyl 1-naphthyl and 1-naphthyl propargyl ethers) were carried out in solvent systems CH_2_Cl_2_/ROH or CHCl_3_/ROH. It was found that addition of alcohol to methylene chloride or chloroform considerably accelerated annulation reactions [70,71]. In solvent systems CH_2_Cl_2_/MeOH or CHCl_3_/MeOH, the reactions proceeded as annulation–methoxylation affording condensed methoxylated heterocycles. In the presence of isopropanol, the reactions were not accompanied by alkoxylation giving condensed halogen-containing products (Scheme 1). The annulation–acetoxylation reaction of selenium dibromide with allyl carvacryl ether in the solvent system CH_2_Cl_2_/acetic acid affording bicylic acetoxy derivative in 98% yield was also developed [71]. Novel condensed functionalized products, 2,3-dihydro-1,4-benzoxaselenine derivatives, bearing methoxy, acetoxy and halogen-containing groups were synthesized in high yields from selenium dihalides and allyl thymyl and allyl carvacryl ethers using this novel methodology (Scheme 1) [70,71].

## 2. Results and Discussion

**Scheme 2:**
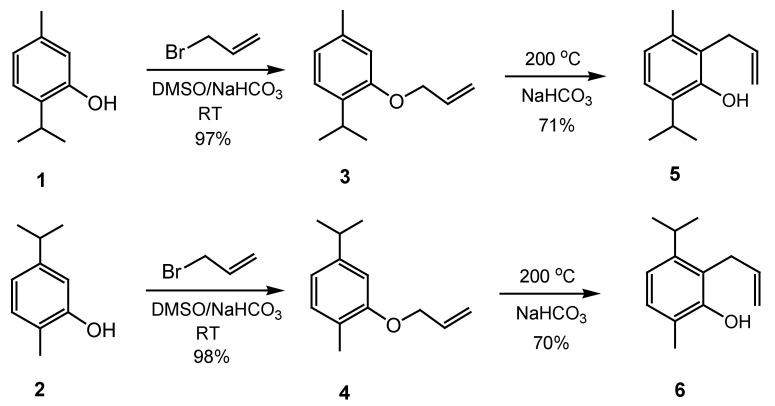
Synthesis of allyl thymol **5** and allyl carvacrol **6** by the reaction of thymol **1** and carvacrol **2** with allyl bromide followed by Claisen rearrangement of compounds **3** and **4**.

The aim of this research is to develop an efficient regioselective synthesis of novel condensed functionalized organochalcogen compounds by chalcogenocyclofunctionalization reactions based on chalcogen halides and natural products thymol (**1**) and carvacrol (**2**). Starting compounds allyl ethers of thymol and carvacrol were prepared by the reaction of thymol and carvacrol with allylbromide in the presence of a base. Usually strong bases as sodium hydroxide or potassium carbonate are often used in alkylation reactions of phenols [72]. We developed efficient green chemistry synthesis of allyl thymyl ether **3** and allyl carvacryl ether **4** in near quantitative yields (97–98%) at room temperature using environmentally tolerant sodium hydrocarbonate (NaHCO_3_, baking soda, E number food additive code is E500) instead of strong bases like alkalis or carbonates (Scheme 2).

The conversion of allyl ethers **3** and **4** to corresponding allyl phenols **5** and **6** was developed using Claisen rearrangement (Scheme 2). It was established that the products **5** and **6** were formed in 75–80% yields when the reaction was carried out by heating allyl ethers **3** and **4** at 200 °C for 2 h in sealed tubes. The reaction is a [3,3]-sigmatropic rearrangement which leads to the formation of new carbon-carbon bond. It was found that an additive of sodium hydrocarbonate increases selectivity of the rearrangement. Pure products **5** and **6** were isolated by column chromatography on silica gel in 70–71% yields (Scheme 2). Minor amounts of allyl ethers **3** and **4** remained unconverted in Claisen rearrangement, however, products **5** and **6** containing a polar hydroxyl group can be easily separated from them.

**Scheme 3:**
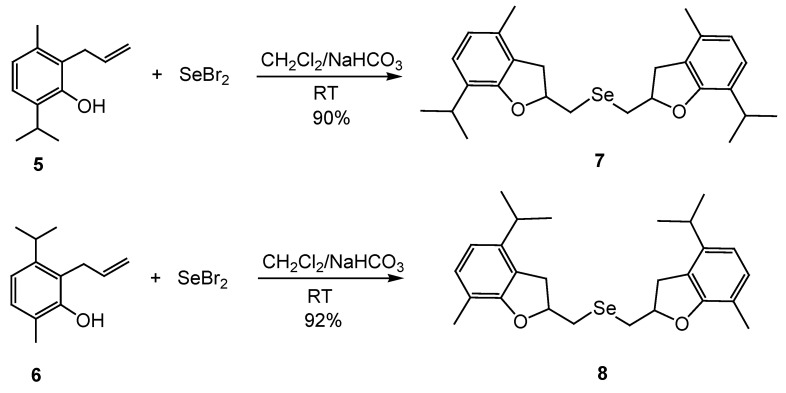
Synthesis of selenides **7** and **8** by the reaction of selenium dibromide with allyl thymol **5** and allyl carvacrol **6**.

We studied the reactions of selenium dihalides with allyl thymol **5** and allyl carvacrol **6** and found that selenium dibromide is more efficient than selenium dichloride in selenocyclofunctionalization. The reactions smoothly proceeded in methylene chloride at room temperature. After completing the first stage (electrophilic addition of selenium dibromide to the double bond of allyl thymol **5** and allyl carvacrol **6**), sodium hydrocarbonate as a mild base was added to the reaction mixture in order to catalyze intramolecular nucleophilic substitution of the bromine atom by the hydroxyl group with the formation of bis[(7-isopropyl-4-methyl-2,3-dihydro-1-benzofuran-2-yl)methyl] and bis[(4-isopropyl-7-methyl-2,3-dihydro-1-benzofuran-2-yl)methyl] selenides **7** and **8** in 90–92% yield (Scheme 3). In the case of using selenium dichloride, the yields of products **7** and **8** were 67–70%.

**Scheme 4:**
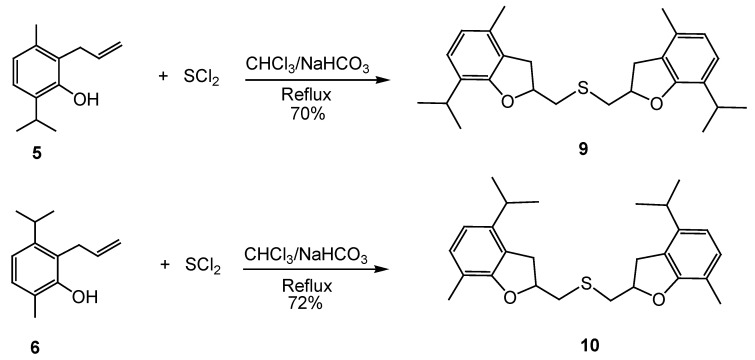
Synthesis of sulfides **9** and **10** by the reaction of sulfur dichloride with allyl thymol **5** and allyl carvacrol **6**.

The reaction of sulfur dichloride with allyl thymol **5** and allyl carvacrol **6** was very sluggish under the same conditions as synthesis of selenium analogues **7** and **8**. Heating under reflux in chloroform was found to be necessary in order to accelerate the reaction. Besides, using a 20% excess of sulfur dichloride is advisable, otherwise a part of allyl thymol **5** and allyl carvacrol **6** stayed unconverted. After heating under reflux, sodium hydrocarbonate was added in order to catalyze intramolecular nucleophilic substitution and the reaction mixture was stirred overnight at room temperature. Bis[(7-isopropyl-4-methyl-2,3-dihydro-1-benzofuran-2-yl)methyl] and bis[(4-isopropyl-7-methyl-2,3-dihydro-1-benzofuran-2-yl)methyl] sulfides **9** and **10** were isolated by column chromatography on silica gel in 70 and 72% yields, respectively (Scheme 4).

**Scheme 5:**
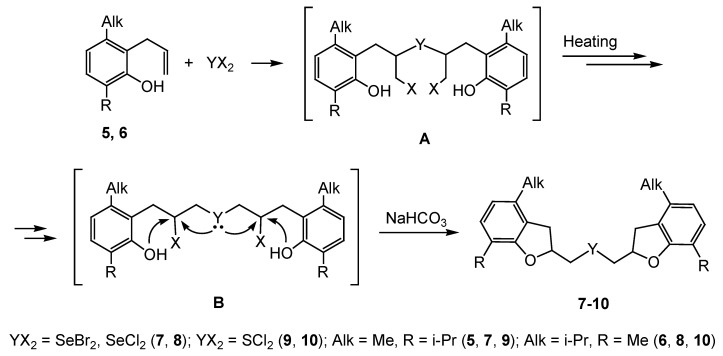
A supposed pathway of the reactions of SeBr_2_, SeCl_2_ and SCl_2_ with allyl thymol **5** and allyl carvacrol **6**.

We suppose that heating accelerates isomerization of primarily formed anti-Markovnikov adducts **A** into Markovnikov products **B** followed by intramolecular nucleophilic substitution with the formation of (2,3-dihydro-1-benzofuran-2-yl)methyl moiety (Scheme 5). It is known that electrophilic addition of sulfenyl halides to 1-alkenes leads predominantly to anti-Markovnikov products [73,74,75]. 

We suppose that similar consequence of transformations (Scheme 5) also takes place in selenocyclofunctionalization reactions (Scheme 3). The isomerization process and intramolecular nucleophilic substitution proceed smoothly at room temperature due to high anchimeric assistance effect of the selenium atom, which activates halogen atoms in β-haloorganyl selenides **B** (Scheme 5) [58]. Previously, we observed the formation of anti-Markovnikov adducts in the reactions of selenium dibromide with 1-alkenes and their isomerization into thermodynamically more stable Markovnikov products at room temperature [76,77]. The bromine atom is better leaving group compared to the chlorine atom in nucleophilic substitution and selenium dibromide is more efficient than selenium dichloride in these reactions.

**Scheme 6:**
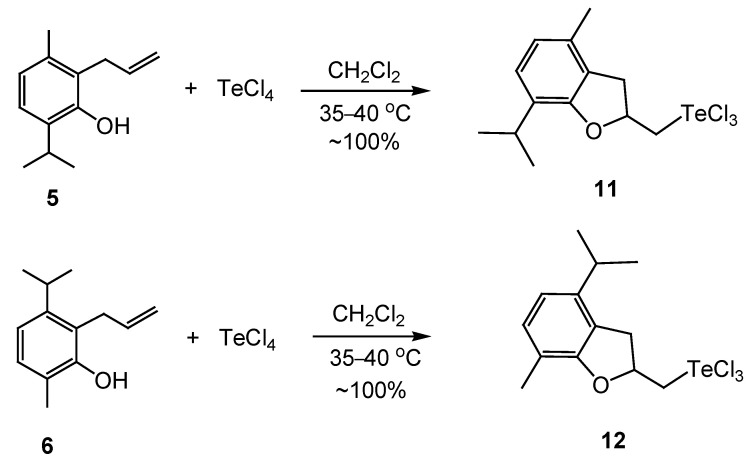
Synthesis of tellanes **11** and **12** by the reaction of tellurium tetrachloride with allyl thymol **5** and allyl carvacrol **6**.

Tellurium tetrachloride and tetrabromide were used in the cyclofunctionalization reactions with allyl thymol **5** and allyl carvacrol **6**, and conditions for efficient selective reactions with each of these tellurium-centered electrophilic reagents were established. We found that the reactions of tellurium tetrachloride with allyl thymol **5** and allyl carvacrol **6** proceeded smoothly in methylene chloride at the temperature 35–40 °C affording trichlorotellanes **11** and **12** bearing the (2,3-dihydro-1-benzofuran-2-yl)methyl moiety in quantitative yields (Scheme 6). 

**Scheme 7:**
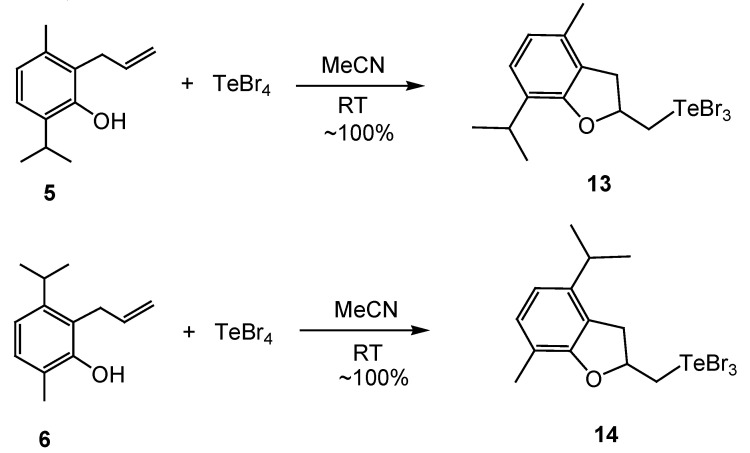
Synthesis of tellanes **13** and **14** by the reaction of tellurium tetrabromide with allyl thymol **5** and carvacrol **6**.

The disadvantage of using tellurium tetrabromide is the low solubility of this reagent in most common organic solvents (chloroform, methylene chloride, benzene, diethyl ether, carbon tetrachloride). However, we found that the reactions of tellurium tetrabromide with allyl thymol **5** and allyl carvacrol **6** can be efficiently carried out in acetonitrile at room temperature affording tribromotellanes **13** and **14** in quantitative yield (Scheme 7). 

The intramolecular substitution is activated by anchimeric assistance of the selenium atom in the reactions with selenium dibromide (Scheme 3), however, in the case of tellurium tetrachloride and tetrabromide (Scheme 6 and Scheme 7), the halogen atom in the addition products is very reactive due to high electron-withdrawing effect of trichlorotellanyl and tribromotellanyl groups.

It is worth noting that reactions of tellurium tetrachloride and tetrabromide with allyl thymol **5** and allyl carvacrol **6** proceeded with high selectivity in a regiospecific manner affording only monoadducts **11**–**14** in quantitative yields (Scheme 6 and Scheme 7).

**Scheme 8:**
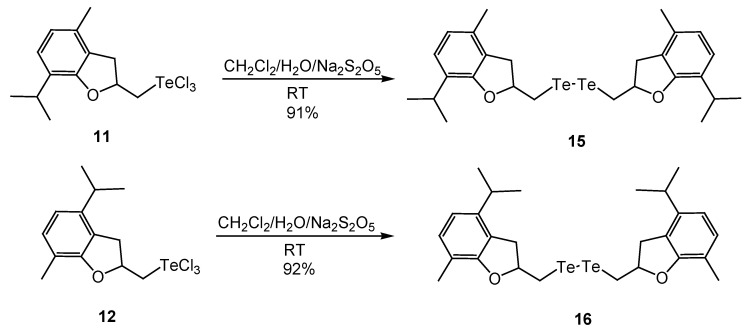
Synthesis of ditellurides **15** and **16** by the reduction of trichlorotellanes **11** and **12** with sodium metabisulfite.

Finally, we obtained functionalized ditellurides **15** and **16** bearing the (2,3-dihydro-1-benzofuran-2-yl)methyl moiety in 91–92% yields by the reduction of trichlorotellanes **11** and **12** with sodium metabisulfite (Scheme 8). The reactions were selectively carried out in the two-phase solvent system of water and methylene chloride at room temperature giving the products **15** and **16**, which did not require additional purification.

The obtained products represent a new family of organochalcogen compounds bearing (7-isopropyl-4-methyl-2,3-dihydro-1-benzofuran-2-yl)methyl and (4-isopropyl-7-methyl-2,3-dihydro-1-benzofuran-2-yl)methyl moieties with promising biological activity.

The structural assignments of the synthesized compounds were made using ^1^H-, ^13^C-, ^77^Se- and ^125^Te-NMR spectroscopy and confirmed by elemental analysis. The compounds **7**–**10**, **15**, **16** consist of two diastereomers (*dl* and *meso*) approximately in an equimolar ratio. Two closely spaced signals which correspond to two diastereomers were observed in the ^77^Se NMR spectra of compounds **7** and **8.**

The signals of the CH_2_TeCl_3_ and CH_2_TeBr_3_ groups in ^13^C-NMR spectra of compounds **11**–**14** manifest themselves in the downfield region (66.0–66.3 and ~61.5 ppm) due to high electron-withdrawing effect of trichlorotellanyl and tribromotellanyl groups. The signals of the CH_2_Te group in ^13^C-NMR spectra of ditellurides **15** and **16** are observed in the upfield region (~11.8 ppm).

The tellurium atom of the CH_2_TeCl_3_ group in ^125^Te NMR spectra of compounds **11** and **12** resonated in the downfield region (1206.3 and 1204.0 ppm) due to the presence of three electronegative chlorine atoms. However, the signals in ^125^Te-NMR spectra of ditellurides **15** and **16,** which were obtained by the reduction of compounds **11** and **12**, were observed in the upfield region (77.9 and 84.0 ppm). 

## 3. Experimental Section

### 3.1. General Information

The ^1^H (400.1 MHz), ^13^C (100.6 MHz), ^77^Se (76.3 MHz) and ^125^Te (126.4 MHz) NMR spectra (see Supplementary Materials) were recorded on a Bruker DPX-400 spectrometer (Bruker BioSpin GmbH, Rheinstetten, Germany) in CDCl_3_ and *d*_6_-DMSO solutions and referred to the residual solvent peaks of CDCl_3_ (δ = 7.27 and 77.16 ppm in ^1^H- and ^13^C-NMR, respectively) and *d*_6_-DMSO (δ = 2.50 and 39.5 ppm in ^1^H- and ^13^C-NMR) or Me_2_Se (^77^Se-NMR, external) and Me_2_Te (^125^Te-NMR, external). Elemental analysis was performed on a Thermo Scientific Flash 2000 Elemental Analyzer (Thermo Fisher Scientific Inc., Milan, Italy). Melting points were determined on a Kofler Hot-Stage Microscope PolyTherm A apparatus (Wagner & Munz GmbH, Munich, Germany). The organic solvents were dried and distilled according to standard procedures. Silica gel (0.06–0.20 mm (70–230 mesh, Alfa Aesar, Heysham, Lancashire, United Kingdom) was used for column chromatography.

### 3.2. Synthesis of Starting Compounds ***5*** and ***6***

*2-Allyl-6-isopropyl-3-methylphenol* (**5**). A mixture of allyl thymyl ether **3** (4 g, 21 mmol) and dry powdered NaHCO_3_ (1 g, 12 mmol) was heated at 200 °C for 2 h in a sealed tube under argon. Compound **5** (2.84 g, 71% yield) was isolated as a colourless liquid by column chromatography on silica gel (eluent: hexane, then hexane/methylene chloride 1:10). 

^1^H-NMR (CDCl_3_): 1.39 (d, 6H, CH_3_CH, *J* = 7 Hz), 2.41 (s, 3H,CH_3_C_Ar_), 3.26–3.35 (m, 1H, CH_3_CH), 3.55–3.58 (m, 2H, CH_2_C_Ar_), 5.10 (s, 1H, OH), 5.21–5.27 (m, 2H, CH_2_=CH), 6.06–6.16 (m, 1H, CH_2_=CH), 6.91 (d, 1H, CH_Ar_, *J* = 7.8 Hz), 7.15 (d, 1H, CH_Ar_, *J* = 7.8 Hz). ^13^C-NMR (CDCl_3_): 19.7 (CH_3_C_Ar_), 22.8 (CH_3_CH), 27.0 (CH_3_CH), 31.3 (CH_2_C_Ar_), 116.0 (CH_2_=CH), 122.5 (CH_Ar_), 123.2 (CH_2_C_Ar_), 123.9 (CH_Ar_), 132.4 (CHC_Ar_), 134.9 (CH_3_C_Ar_), 135.7 (CH_2_=CH), 151.6 (C_Ar_OH). Anal. calcd for C_13_H_18_O (190.28): C 82.06, H 9.53%. Found: C 82.31, H 9.38%.

*2-Allyl-3-isopropyl-6-methylphenol* (**6**) was obtained as a colourless liquid (2.80 g, 70% yield) from allyl carvacryl ether **4** (4 g, 21 mmol) and dry powdered NaHCO_3_ (1 g, 12 mmol) by heating the mixture at 200 °C for 2 h in a sealed tube followed by isolation by column chromatography on silica gel (eluent: hexane, then hexane/methylene chloride 1:10). ^1^H-NMR (CDCl_3_): 1.34 (d, 6H, CH_3_CH, *J* = 7 Hz), 2.34 (s, 3H,CH_3_C_Ar_), 3.19–3.25 (m, 1H, CH_3_CH), 3.60–3.63 (m, 2H, CH_2_C_Ar_), 5.03 (s, 1H, OH), 5.16–5.25 (m, 2H, CH_2_=CH), 6.08–6.18 (m, 1H, CH_2_=CH), 6.95 (d, 1H, CH_Ar_, *J* = 7.9 Hz), 7.14 (d, 1H, CH_Ar_, *J* = 7.9 Hz) ^13^C-NMR (CDCl_3_): 15.9 (CH_3_C_Ar_) 24.1 (CH_3_CH), 29.3 (CH_3_CH), 30.1 (CH_2_C_Ar_), 115.8 (CH_2_=CH), 117.3 (CH_Ar_), 121.1 (CH_3_C_Ar_), 122.1 (CH_2_C_Ar_), 128.9 (CH_Ar_), 136.6 (CH_2_=CH), 146.1 (CHC_Ar_), 152.4 (C_Ar_OH). Anal. calcd for C_13_H_18_O (190.28): C 82.06, H 9.53%. Found: C 82.24, H 9.67%.

### 3.3. Synthesis of Products ***7***–***16***

*Bis[(7-isopropyl-4-methyl-2,3-dihydro-1-benzofuran-2-yl)methyl] selenide* (**7**). A solution of selenium dibromide (0.5 mmol) was prepared from selenium (0.04 g, 0.5 mmol) and bromine (0.08 g, 0.5 mmol) in methylene chloride (2 mL). The obtained solution of selenium dibromide (0.5 mmol) was added dropwise to a solution of allyl thymol **5** (0.19 g, 1 mmol) in methylene chloride (10 mL). The mixture was stirred for 7 h at room temperature and NaHCO_3_ (0.17 g, 2 mmol) was added. The mixture was stirred overnight (14 h) at room temperature. The mixture was filtered and the solvent was removed by a rotary evaporator. The residue was subjected to column chromatography on silica gel (eluent: hexane, then hexane/chloroform 1:10). Compound **7** (0.206 g, 90% yield) was isolated as a colourless viscous oil. ^1^H-NMR (CDCl_3_): 1.26 (d, 12H, CH_3_CH, *J* = 6.8 Hz), 2.19 (d, 6H,CH_3_C_Ar_, *J* = 7.2 Hz), 2.83–2.91 (m, 2H, CH_3_CH), 2.93–3.06 (m, 4H, CH_2_Se, CH_2_C_Ar_), 3.10–3.20 (m, 2H, CH_2_Se), 3.35–3.43 (m, 2H, CH_2_C_Ar_), 5.02–5.09 (m, 2H, CHO), 6.71 (d, 2H, CH_Ar_, *J* = 7.7 Hz), 6.95 (d, 2H, CH_Ar_, *J* = 7.7 Hz). ^13^C-NMR (CDCl_3_): 15.1 (CH_3_C_Ar_), 15.1 (CH_3_C_Ar_), 23.0 (CH_3_CH), 23.0 (CH_3_CH), 29.7 (CH_2_Se), 30.0 (CH_2_Se), 31.4 (CH_3_CH), 35.0 (CH_2_C_Ar_), 82.5 (CHO), 117.0 (CH_Ar_), 123.2 (CH_2_C_Ar_), 123.2 (CH_3_C_Ar_), 129.7 (CH_Ar_), 143.0 (CHC_Ar_), 157.4 (C_Ar_O). ^77^Se-NMR (CDCl_3_): 103.1, 104.4. Anal. calcd for C_26_H_34_O_2_Se (457.51): C 68.26, H 7.49, Se 17.26%. Found: C 68.48, H 7.64, Se 17.49%.

*Bis[(4-isopropyl-7-methyl-2,3-dihydro-1-benzofuran-2-yl)methyl] selenide* (**8**) was obtained as a colourless viscous oil (0.211 g, 92% from selenium dibromide (0.5 mmol) and allyl carvacrol **6** (0.19 g, 1 mmol) in methylene chloride under the same conditions as compound **7**. ^1^H-NMR (CDCl_3_): 1.27 (d, 12H, CH_3_CH, *J* = 6.8 Hz), 2.25 (s, 6H,CH_3_C_Ar_), 2.94–3.02 (m, 4H, CH_3_CH, CH_2_Se), 3.06–3.19 (m, 4H, CH_2_Se, CH_2_C_Ar_), 3.29–3.36 (m, 2H, CH_2_C_Ar_), 5.05–5.09 (m, 2H, CHO), 6.69 (d, 2H, CH_Ar_, *J* = 7.6 Hz), 6.97 (d, 2H, CH_Ar_, *J* = 7.6 Hz). ^13^C-NMR (100 MHz,CDCl_3_): 18.7 (CH_3_Ar), 22.6 (CH_3_CH), 28.2 (CH_3_CH), 29.7 (CH_2_Se), 30.0 (CH_2_Se), 35.1 (CH_2_Ar), 82.5 (CHO), 121.6 (HC_Ar_), 124.9 (CH_2_C_Ar_), 125.1 (HC_Ar_), 127.9 (CHC_Ar_), 132.0 (CH_3_C_Ar_), 156.4 (OC_Ar_). ^77^Se NMR (,CDCl_3_): 106.4, 108.2. Anal. calcd for C_26_H_34_O_2_Se (457.51): C 68.26, H 7.49, Se 17.26%. Found: C 67.98, H 7.65, Se 17.47%.

*Bis[(7-isopropyl-4-methyl-2,3-dihydro-1-benzofuran-2-yl)methyl] sulfide* (**9**). A solution of sulfur dichloride (0.62 g, 0.6 mmol) in methylene chloride (2 mL) was added dropwise to a solution of allyl thymol **5** (0.19 g, 1 mmol) in methylene chloride (10 mL). The mixture was stirred for 1 h at room temperature and refluxed for 7 h. NaHCO_3_ (0.25 g, 3 mmol) was added and the mixture was stirred overnight (14 h) at room temperature. The mixture was filtered and the solvent was removed by a rotary evaporator. The residue was subjected to column chromatography on silica gel (eluent: hexane, then hexane/chloroform 1:10). Compound **9** (0.144 g, 70% yield) was isolated as a colourless viscous oil. ^1^H-NMR (CDCl_3_): 1.25 (d, 12H, CH_3_CH, *J* = 6.6 Hz), 2.24 (s, 6H,CH_3_C_Ar_), 2.88–2.96 (m, 2H, CH_3_CH), 2.97–3.02 (m, 2H, CH_2_S), 3.05–3.08 (m, 2H, CH_2_C_Ar_), 3.09–3.16 (m, 2H, CH_2_S), 3.25–3.31 (m, 2H, CH_2_C_Ar_), 4.98–5.06 (m, 2H, CHO), 6.68 (d, 2H, CH_Ar_, *J* = 7.9 Hz), 6.96 (d, 2H, CH_Ar_, *J* = 7.9 Hz). ^13^C-NMR (CDCl_3_): 18.7 (CH_3_C_Ar_), 22.6 (CH_3_CH), 28.2 (CH_3_CH), 34.3 (CH_2_C_Ar_), 37.7 (CH_2_S), 38.1 (CH_2_S), 82.1 (CHO), 121.6 (CH_Ar_), 124.8 (CH_2_C_Ar_), 125.1 (CH_Ar_), 127.9 (CHC_Ar_), 132.1 (CH_3_C_Ar_), 156.4 (C_Ar_O). Anal. calcd for C_26_H_34_O_2_S (410.61): C 76.05, H 8.35, S 7.81%. Found: C 76.28, H 8.19, S 7.65%.

*Bis[(4-isopropyl-7-methyl-2,3-dihydro-1-benzofuran-2-yl)methyl] sulfide* (**10**) was obtained as a colourless viscous oil (0.148 g, 72% yield) from sulfur dichloride (0.62 g, 0.6 mmol) and allyl carvacrol **6** (0.19 g, 1 mmol) in methylene chloride under the same conditions as compound **9.** ^1^H-NMR (CDCl_3_): 1.23 (d, 12H, CH_3_CH, *J* = 6.5 Hz), 2.16 (d, 6H,CH_3_C_Ar_, *J* = 10.8 Hz), 2.81–2.95 (m, 4H, CH_3_CH, CH_2_S), 2.99–3.06 (m, 2H, CH_2_C_Ar_), 3.09–3.15 (m, 2H, CH_2_S), 3.30–3.36 (m, 2H, CH_2_C_Ar_), 4.95–5.02 (m, 2H, CHO), 6.68 (d, 2H, CH_Ar_, *J* = 7.5 Hz), 6.91 (d, 2H, CH_Ar_, *J* = 7.5 Hz). ^13^C-NMR (CDCl_3_): 15.1 (CH_3_C_Ar_), 15.1 (CH_3_C_Ar_), 23.0 (CH_3_CH), 23.0 (CH_3_CH), 31.5 (CH_2_C_Ar_), 34.3 (CH_3_CH), 37.8 (CH_2_S), 38.2 (CH_2_S), 82.3 (CHO), 117.0 (CH_3_C_Ar_), 117.1 (CH_Ar_), 123.2 (CH_2_C_Ar_), 129.7 (CH_Ar_), 143.0 (CHC_Ar_), 157.4 (C_Ar_O). Anal. calcd for C_26_H_34_O_2_S (410.61): C 76.05, H 8.35, S 7.81%. Found: C 75.91, H 8.54, S 7.76%.

*Trichloro[(7-isopropyl-4-methyl-2,3-dihydro-1-benzofuran-2-yl)methyl]-λ^4^-tellane* (**11**). A solution of allyl thymol **5** (0.19 g, 1 mmol) in methylene chloride (2 mL) was added dropwise to a stirred mixture of tellurium tetrachloride (0.27 g, 1 mmol) and methylene chloride (10 mL). The obtained mixture was stirred at room temperature for 2 h and at 35–40 °C for 6 h. The solvent was removed by a rotary evaporator and the residue was dried in vacuum. Compound **11** (0.423 g, quantitative yield) was isolated as a grey viscous oil.

^1^H-NMR (CDCl_3_): 1.20 (d, 3H, CH_3_CH, *J* = 7.1 Hz), 1.29 (d, 3H, CH_3_CH, *J* = 7.1 Hz), 2.28 (s, 3H,CH_3_C_Ar_), 2.97–3.04 (m, 1H, CH_3_CH), 3.26–3.33 (m, 1H, CH_2_C_Ar_), 3.51–3.57 (m, 1H, CH_2_C_Ar_), 4.54–4.65 (m, 2H, CH_2_Te), 5.85–5.93 (m, 1H, CHO), 6.85 (d, 1H, CH_Ar_, *J* = 7.7 Hz), 7.08 (d, 1H, CH_Ar_, *J* = 7.7 Hz). ^13^C-NMR (DMSO-*d*_6_): 18.5 (CH_3_C_Ar_), 22.4 (CH_3_CH), 22.5 (CH_3_CH), 27.8 (CH_3_CH), 35.3 (CH_2_C_Ar_), 66.3 (CH_2_Te), 79.2 (CHO), 121.6 (CH_Ar_), 124.7 (CH_2_C_Ar_), 124.8 (CH_Ar_), 127.1 (CHC_Ar_), 131.7 (CH_3_C_Ar_), 155.4 (C_Ar_O). ^125^Te-NMR (DMSO-*d*_6_): 1206.3. Anal. calcd for C_13_H_17_OCl_3_Te (423.23): C 36.79, H 4.05, Cl 25.13, Te 30.15%. Found: C 36.96, H 3.98, Cl 25.29, Te 29.98%.

*Trichloro[(4-isopropyl-7-methyl-2,3-dihydro-1-benzofuran-2-yl)methyl]-λ^4^-tellane(***12**) was obtained as a colourless viscous oil (0.423 g, quantitative yield) from tellurium tetrachloride (0.27 g, 1 mmol) and allyl carvacrol **6** (0.19 g, 1 mmol) in methylene chloride under the same conditions as compound **11.** ^1^H-NMR (CDCl_3_): 1.20 (d, 3H, CH_3_CH, *J* = 6.9 Hz), 1.30 (d, 3H, CH_3_CH, *J* = 6.9 Hz), 2.22 (s, 3H,CH_3_C_Ar_), 2.88–2.96 (m, 1H, CH_3_CH), 3.31–3.38 (m, 1H, CH_2_C_Ar_), 3.54–3.62 (m, 1H, CH_2_C_Ar_), 4.50–4.61 (m, 2H, CH_2_Te), 5.86–5.93 (m, 1H, CHO), 6.90 (d, 1H, CH_Ar_, *J* = 7.8 Hz), 7.08 (d, 1H, CH_Ar_, *J* = 7.8 Hz). ^13^C-NMR (DMSO-*d*_6_): 15.0 (CH_3_C_Ar_), 22.7 (CH_3_CH), 23.0 (CH_3_CH), 30.0 (CH_3_CH), 35.1 (CH_2_C_Ar_), 66.0 (CH_2_Te), 79.2 (CHO), 116.2 (CH_Ar_), 117.1 (CH_2_C_Ar_), 122.9 (CH_3_C_Ar_), 129.3 (CH_Ar_), 142.6 (CHC_Ar_), 156.4 (C_Ar_O). ^125^Te-NMR (DMSO-*d_6_*): 1204.0. Anal. calcd for C_13_H_17_OCl_3_Te (423.23): C 36.89, H 4.15, Cl 25.13, Te 30.15%. Found: C 37.08, H 4.03, Cl 24.92, Te 30.32%.

*Tribromo[(7-isopropyl-4-methyl-2,3-dihydro-1-benzofuran-2-yl)methyl]-λ^4^-tellane(***13**). A solution of allyl thymol **5** (0.19 g, 1 mmol) in acetonitrile (2 mL) was added to a stirred mixture of tellurium tetrabromide (0.27 g, 1 mmol) in acetonitrile (8 mL). The obtained mixture was stirred at room temperature overnight (16 h). The solvent was removed by a rotary evaporator and the residue was dried in vacuum. Compound **13** (0.423 g, quantitative yield) was obtained as a light orange powder, mp 61–63 °C (decomp.). ^1^H-NMR (CDCl_3_): 1.03 (d, 3H, CH_3_CH, *J* = 6.8 Hz), 1.05 (d, 3H, CH_3_CH, *J* = 6.8 Hz), 2.04 (s, 3H,CH_3_C_Ar_), 2.82–2.92 (m, 1H, CH_3_CH), 3.09–3.16 (m, 1H, CH_2_C_Ar_), 3.27–3.33 (m, 1H, CH_2_C_Ar_), 3.87–3.96 (m, 1H, CH_2_Te), 4.04–4.15 (m, 1H, CH_2_Te), 5.48–5.55 (m, 1H, CHO), 6.46 (d, 1H, CH_Ar_, *J* = 7.9 Hz), 6.73 (d, 1H, CH_Ar_, *J* = 7.9 Hz). ^13^C-NMR (CDCl_3_/DMSO-*d*_6_): 18.1 (CH_3_C_Ar_), 21.9 (CH_3_CH), 27.4 (CH_3_CH), 34.9 (CH_2_C_Ar_), 61.5 (CH_2_Te), 79.5 (CHO), 121.0 (CH_Ar_), 124.2 (CH_2_C_Ar_), 124.2 (CH_Ar_), 127.1 (CHC_Ar_), 131.5 (CH_3_C_Ar_), 155.2 (C_Ar_O). Anal. calcd for C_13_H_17_OBr_3_Te (556.58): C 28.05, H 3.08, Br 43.07, Te 22.93%. Found: C 27.78, H 2.99, Br 43.34, Te 23.17%.

*Tribromo[(4-isopropyl-7-methyl-2,3-dihydro-1-benzofuran-2-yl)methyl]*-λ*^4^-tellane(***14**) (0.423 g, quantitative yield) was obtained as a light orange powder, mp 59–61 °C (decomp.) from tellurium tetrabromide (0.27 g, 1 mmol) and allyl carvacrol **6** (0.19 g, 1 mmol) in acetonitrile under the same conditions as compound **13.** ^1^H-NMR (CDCl_3_): 0.95 (d, 3H, CH_3_CH, *J* = 6.8 Hz), 0.96 (d, 3H, CH_3_CH, *J* = 6.8 Hz), 1.89 (s, 3H,CH_3_C_Ar_), 2.56–2.67 (m, 1H, CH_3_CH), 3.20–3.26 (m, 2H, CH_2_Te) 3.81–3.88 (m, 1H, CH_2_C_Ar_), 3.99–4.03 (m, 1H, CH_2_C_Ar_), 5.38–5.45 (m, 1H, CHO), 6.40 (d, 1H, CH_Ar_, *J* = 7.8 Hz), 6.63 (d, 1H, CH_Ar_, *J* = 7.8 Hz). ^13^C-NMR (CDCl_3_/DMSO-*d*_6_): 14.5 (CH_3_C_Ar_), 22.2 (CH_3_CH), 22.4 (CH_3_CH), 30.6 (CH_3_CH), 34.5 (CH_2_C_Ar_), 61.5 (CH_2_Te), 79.6 (CHO), 116.0 (CH_Ar_), 116.4 (CH_2_C_Ar_), 122.5 (CH_3_C_Ar_), 128.7 (CH_Ar_), 142.5 (CHC_Ar_), 156.2 (C_Ar_O). Anal. calcd for C_13_H_17_OBr_3_Te (556.58): C 28.05, H 3.08, Br 43.07, Te 22.93%. Found: C 28.16, H 3.13, Br 43.29, Te 23.14%.

*Bis[(7-isopropyl-4-methyl-2,3-dihydro-1-benzofuran-2-yl)methyl] ditelluride(***15**). A solution of Na_2_S_2_O_5_ (1.2 g, 6.3 mmol) in water (8 mL) was added to a stirred mixture of trichlorotellane **11** (0.423 g, 1 mmol) in methylene chloride (10 mL). The obtained mixture was stirred at room temperature for 6 h. The organic layer was separated and the aqueous phase was extracted by methylene chloride (8 mL). The combined organic phase was dried over CaCl_2_, the solvent was removed by a rotary evaporator and the residue was dried in vacuum. Compound **15** (0.288 g, 91% yield) was obtained as a dark orange viscous oil. ^1^H-NMR (CDCl_3_): 1.15 (d, 12H, CH_3_CH, *J* = 6.9 Hz), 2.11 (s, 6H,CH_3_C_Ar_), 2.76–2.83 (m, 2H, CH_2_C_Ar_), 2.89–2.98 (m, 2H, CH_3_CH), 3.18–3.24 (m, 2H, CH_2_C_Ar_), 3.42–3.47 (m, 2H, CH_2_Te), 3.54–3.60 (m, 2H, CH_2_Te), 4.83–4.90 (m, 2H, CHO), 6.55 (d, 2H, CH_Ar_, *J* = 7.3 Hz), 6.83 (d, 2H, CH_Ar_, *J* = 7.3 Hz). ^13^C-NMR (CDCl_3_): 11.8 (CH_2_Te), 18.6 (CH_3_C_Ar_), 22.3 (CH_3_CH), 22.5 (CH_3_CH), 28.2 (CH_3_CH), 35.6 (CH_2_C_Ar_), 83.7 (CHO), 121.5 (CH_Ar_), 124.8 (CH_2_C_Ar_), 125.0 (CH_Ar_), 127.8 (CHC_Ar_), 131.9 (CH_3_C_Ar_), 156.2 (C_Ar_O). ^125^Te-NMR (100 MHz, CDCl_3_): 77.9. Anal. calcd for C_26_H_34_O_2_Te_2_ (633.75): C 49.27, H 5.41, Te 40.27%. Found: C 49.49, H 5.46, Te 40.08%.

*Bis[(4-isopropyl-7-methyl-2,3-dihydro-1-benzofuran-2-yl)methyl] ditelluride (***16**) was obtained as a dark orange viscous oil (0.291 g, 92% yield) from trichlorotellane **12** (0.423 g, 1 mmol) and Na_2_S_2_O_5_ (1.2 g, 6.3 mmol) in the two-phase solvent system of water (8 mL) and methylene chloride (10 mL). ^1^H-NMR (CDCl_3_): 1.14 (d, 12H, CH_3_CH, *J* = 7.0 Hz), 2.08 (s, 6H,CH_3_C_Ar_), 2.71–2.78 (m, 2H, CH_3_CH), 2.83–2.89 (m, 2H, CH_2_C_Ar_), 3.25–3.32 (m, 2H, CH_2_C_Ar_), 3.40–3.48 (m, 2H, CH_2_Te), 3.56–3.63 (m, 2H, CH_2_Te), 4.84–4.91 (m, 2H, CHO), 6.59 (d, 2H, CH_Ar_, *J* = 7.7 Hz), 6.83 (d, 2H, CH_Ar_, *J* = 7.7 Hz). ^13^C-NMR (CDCl_3_): 11.8 (CH_2_Te), 15.1 (CH_3_C_Ar_), 23.0 (CH_3_CH), 23.0 (CH_3_CH), 31.4 (CH_3_CH), 35.7 (CH_2_C_Ar_), 84.0 (CHO), 117.1 (CH_2_C_Ar_), 123.2 (CH_3_C_Ar_), 128.4 (CH_Ar_), 129.8 (CH_Ar_), 143.0 (CHC_Ar_), 157.4 (C_Ar_O). ^125^Te-NMR (100 MHz, CDCl_3_): 84.0. Anal. calcd for C_26_H_34_O_2_Te_2_ (633.75): C 49.27, H 5.41, Te 40.27%. Found: C 49.51, H 5.58, Te 40.53%.

## 4. Conclusions

There are no works in the literature studying chalcogenocyclofunctionalization reactions of sulfur, selenium and tellurium halides on the same substrates and comparing the reactivity of these electrophilic reagents. The present work described regioselective synthesis of novel functionalized condensed organochalcogen compounds by chalcogeno-cyclofunctionalization reactions based on chalcogen halides and natural products thymol and carvacrol. The conditions for efficient chalcogenocyclofunctionalization by reactions of selenium dihalides, sulfur dichloride and tellurium tetrahalides with allyl thymol and allyl carvacrol were found. Attempts were made to reveal the distinguishing features of each reaction and to compare the reactivity of sulfur, selenium and tellurium halides in chalcogenocyclofunctionalization.

The reactions of tellurium tetrachloride and tetrabromide with allyl thymol and allyl carvacrol proceeded with high selectivity in a regiospecific manner affording monoadducts in quantitative yield.

The obtained products represent a new family of organochalcogen compounds bearing (7-isopropyl-4-methyl-2,3-dihydro-1-benzofuran-2-yl)methyl and (4-isopropyl-7-methyl-2,3-dihydro-1-benzofuran-2-yl)methyl moieties with promising biological activity. 

## Data Availability

Data is available in this article and supplementary information.

## Supplementary Materials

The following are available online: examples of the NMR spectra of the obtained compounds.

## Sample Availability

Samples of the compounds are not available from the authors.
