# Supplementary material for: A Regioselective Synthesis of Novel Functionalized Organochalcogen Compounds by Chalcogenocyclofunctionalization Reactions Based on Chalcogen Halides and Natural Products
Source: Molecules. 2021 Jun 18;26(12):3729. doi: 10.3390/molecules26123729 (PMC8234429; doi:10.3390/molecules26123729)
Supplement: Supplementary file 1 [file molecules-26-03729-s001.zip › molecules-1263778-supplementary.pdf]

Supplementary Materials

A Regioselective Synthesis of Novel Functionalized Organochalcogen Compounds by Chalcogenocyclofunctionalization Reactions Based on Chalcogen Halides and Natural Products

Maxim V. Musalov, Vladimir A. Potapov*, Vladimir A. Yakimov, Maria V. Musalova, Arkady A. Maylyan, Sergey V. Zinchenko and Svetlana V. Amosova

A. E. Favorsky Irkutsk Institute of Chemistry, Siberian Division of The Russian Academy of Sciences, 1 Favorsky Str., Irkutsk 664033, Russia
musalov_maxim@irioch.irk.ru (M.V.M.); yakimov@irioch.irk.ru (V.A.Y.); musalova@irioch.irk.ru (M.V.M.); maylyan@irioch.irk.ru (A.A.M.); svz@irioch.irk.ru (S.V.Z.); amosova@irioch.irk.ru (S.V.A.)
* Correspondence: v.a.potapov@mail.ru

Table of Contents

Experimental (General Information) 2

Synthesis of compounds 3 and 4 3

Examples of NMR Spectra 4-14

Experimental (General Information)

The ^{1}H (400.1 MHz), ^{13}C (100.6 MHz), ^{77}Se (76.3 MHz) and ^{125}Te (126.4 MHz) NMR spectra (see Supplementary Materials) were recorded on a Bruker DPX-400 spectrometer (Bruker BioSpin GmbH, Rheinstetten, Germany) in $CDCl_3$ and ***d***$_6$-DMSO solutions and referred to the residual solvent peaks of $CDCl_3$ (δ = 7.27 and 77.16 ppm in ^{1}H- and ^{13}C-NMR, respectively) and ***d***$_6$-DMSO (δ = 39.5 ppm in ^{13}C-NMR) or Me_2Se (^{77}Se-NMR, external) and Me_2Te (^{125}Te-NMR, external). Elemental analysis was performed on a Thermo Scientific Flash 2000 Elemental Analyzer (Thermo Fisher Scientific Inc., Milan, Italy). Melting points were determined on a Kofler Hot-Stage Microscope PolyTherm A apparatus (Wagner & Munz GmbH, Munich, Germany). The organic solvents were dried and distilled according to standard procedures. Silica gel (Alfa Aesar, 0.06–0.20 mm (70–230 mesh) was used for column chromatography.

Synthesis of compounds 3 and 4

2-(Allyloxy)-1-isopropyl-4-methylbenzene (3). Sodium hydrocarbonate (5 g, 0.06 mol) was added to a solution of thymol (7.5 g, 0.05 mol) in DMSO (25 mL) and the mixture was stirred for 1 h at room temperature. A solution of allyl bromide (7.26 g, 0.06 mol) in DMSO (10 mL) was then added, and the mixture was stirred overnight (15 h) at room temperature. The mixture was diluted with water (100 mL) and extracted with hexane (3×25 mL). The combined organic phase was washed with a 5% aqueous solution of $NaHCO_3$ (10 mL) and water (10 mL) and dried over calcium chloride. After removing the solvent on a rotary evaporator, the residue was dried in vacuum giving ether 3 (9.22 g, 97%) as a colorless liquid.

2-(Allyloxy)-4-isopropyl-1-methylbenzene (4) was obtained as a colourless liquid (9.32 g, 98%) from carvacrol (7.5 g, 0.05 mol) and allyl bromide (7.26 g, 0.06 mol) in DMSO in the presence of sodium hydrocarbonate under the same conditions as compound 3.

The spectral characteristics of obtained compounds 3 and 4 correspond to the literature data: Trivedi, R. and Tunge, J.A., Regioselective Iron-catalyzed decarboxylative allylic etherification. ***Org. Lett.***, 2009, ***11***, 5650. https://doi.org/10.1021/ol902291z

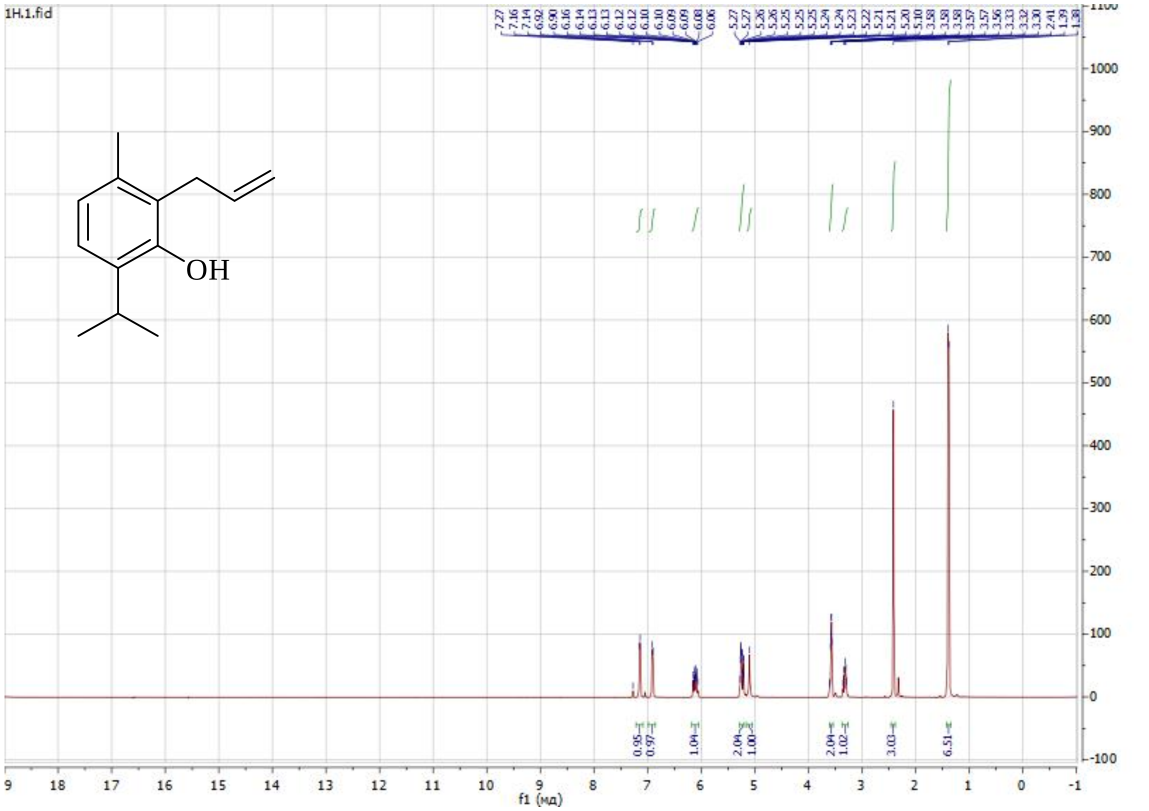

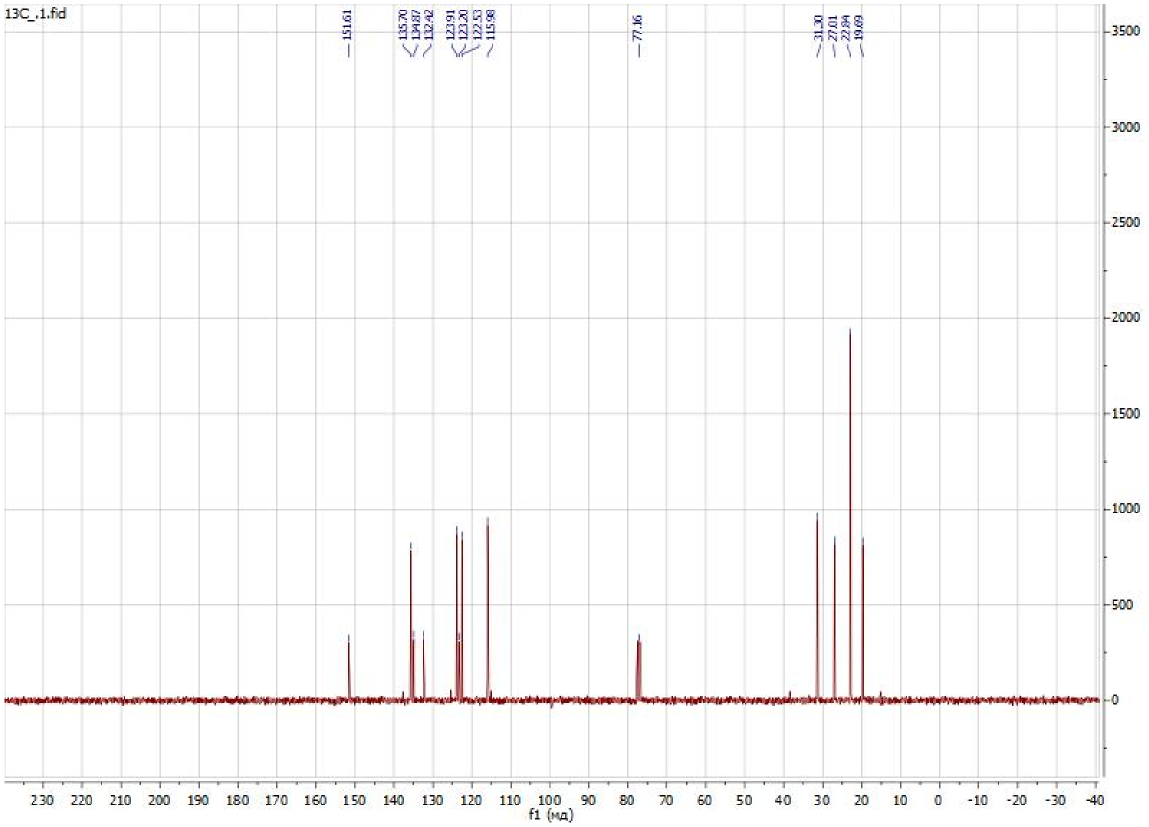

^{1}H- and ^{13}C-NMR spectra of compound 5

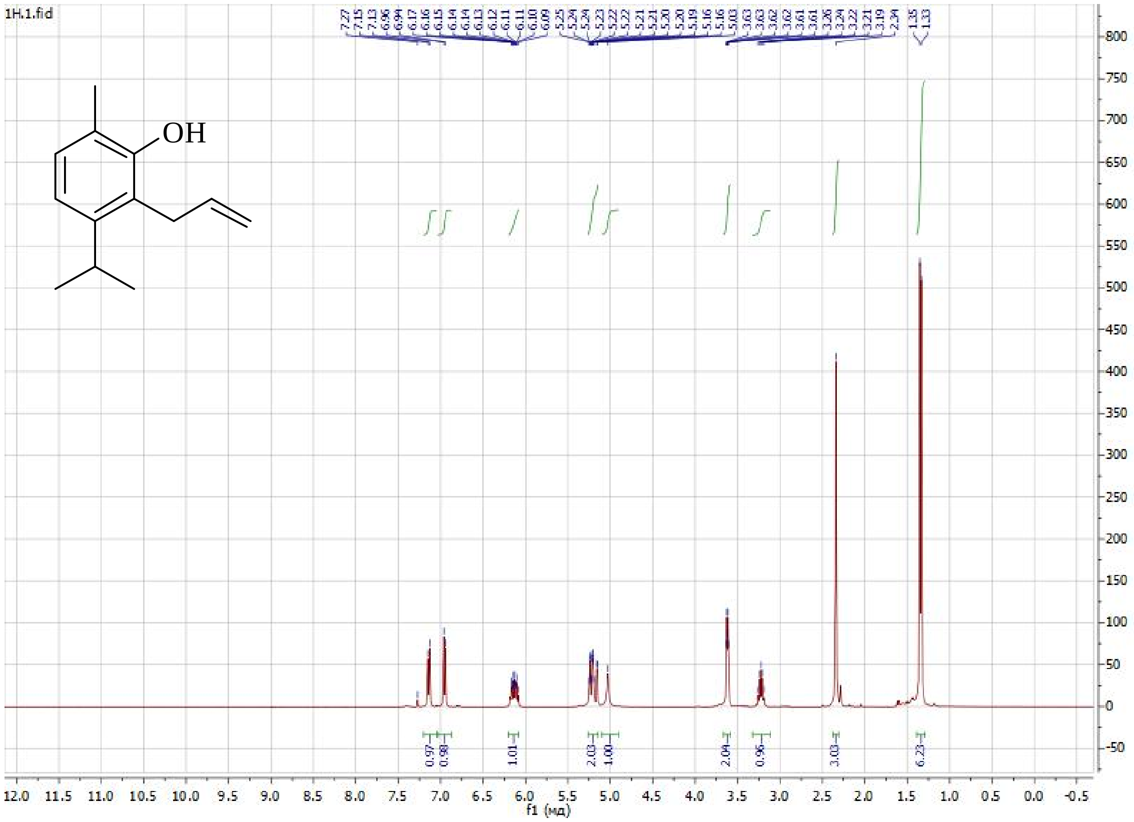

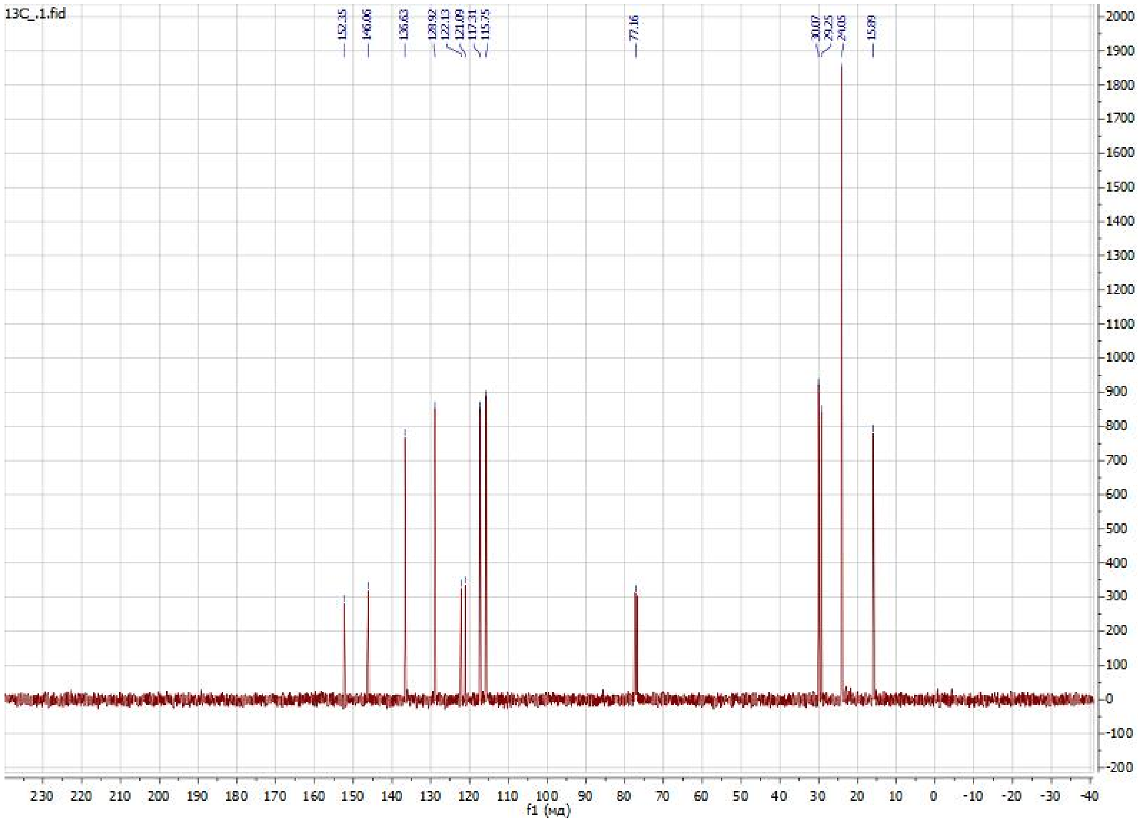

^{1}H- and ^{13}C-NMR spectra of compound 6

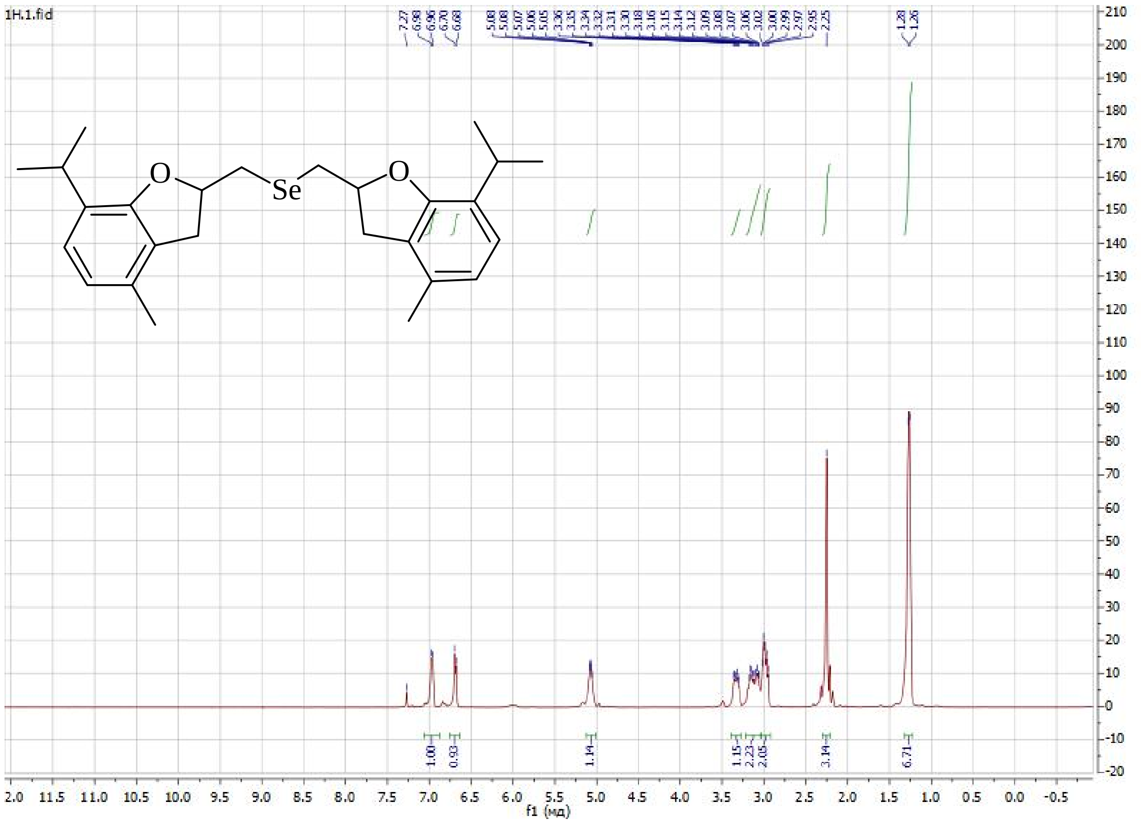
1H.1.fid
O
Se
O
7.27
6.98
6.96
6.70
6.68
5.08
5.08
5.07
5.06
5.05
3.36
3.35
3.34
3.32
3.31
3.30
3.18
3.16
3.15
3.14
3.12
3.09
3.08
3.07
3.06
3.02
3.00
2.99
2.97
2.95
2.25
1.28
1.26
1.00
0.93
1.14
1.15
2.23
2.05
3.14
6.71
f1 (мд)

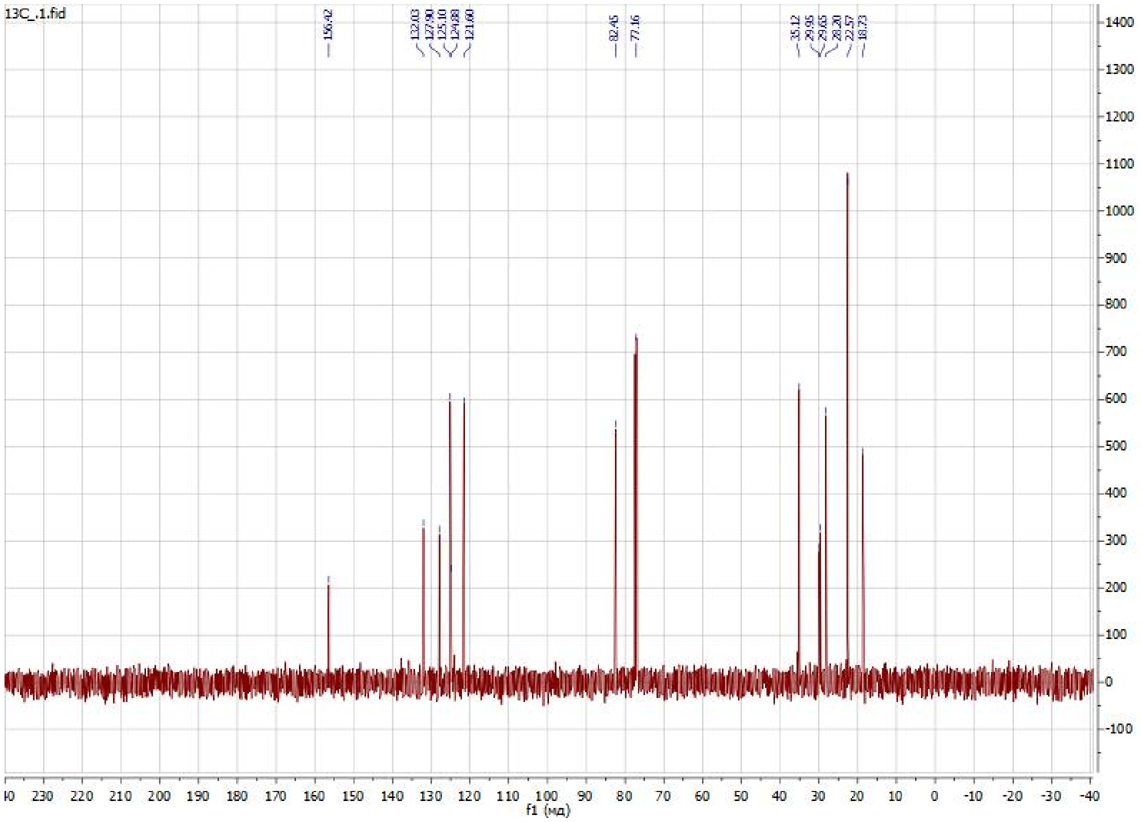
13C_.1.fid
156.42
132.03
127.90
125.10
124.88
121.60
82.45
77.16
35.12
29.95
29.65
28.20
22.57
18.73
f1 (мд)

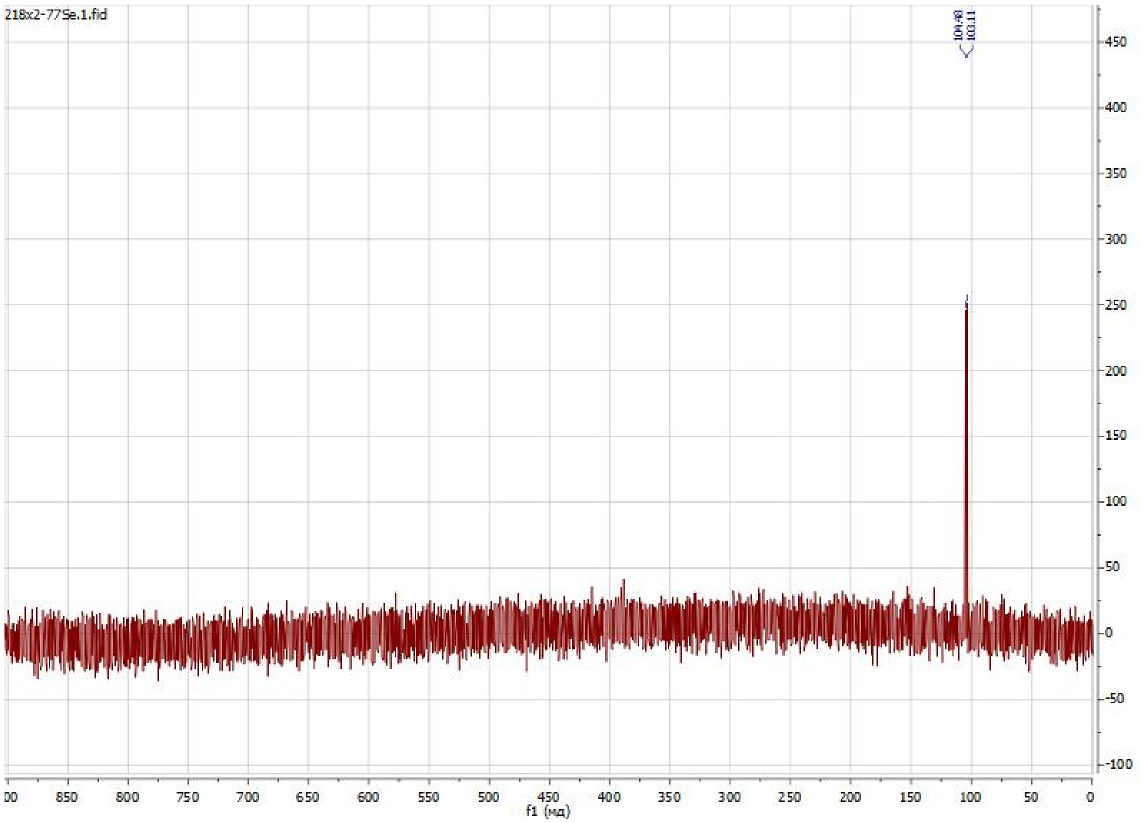

^{1}H-, ^{13}C- and ^{77}Se NMR spectra of compound 7

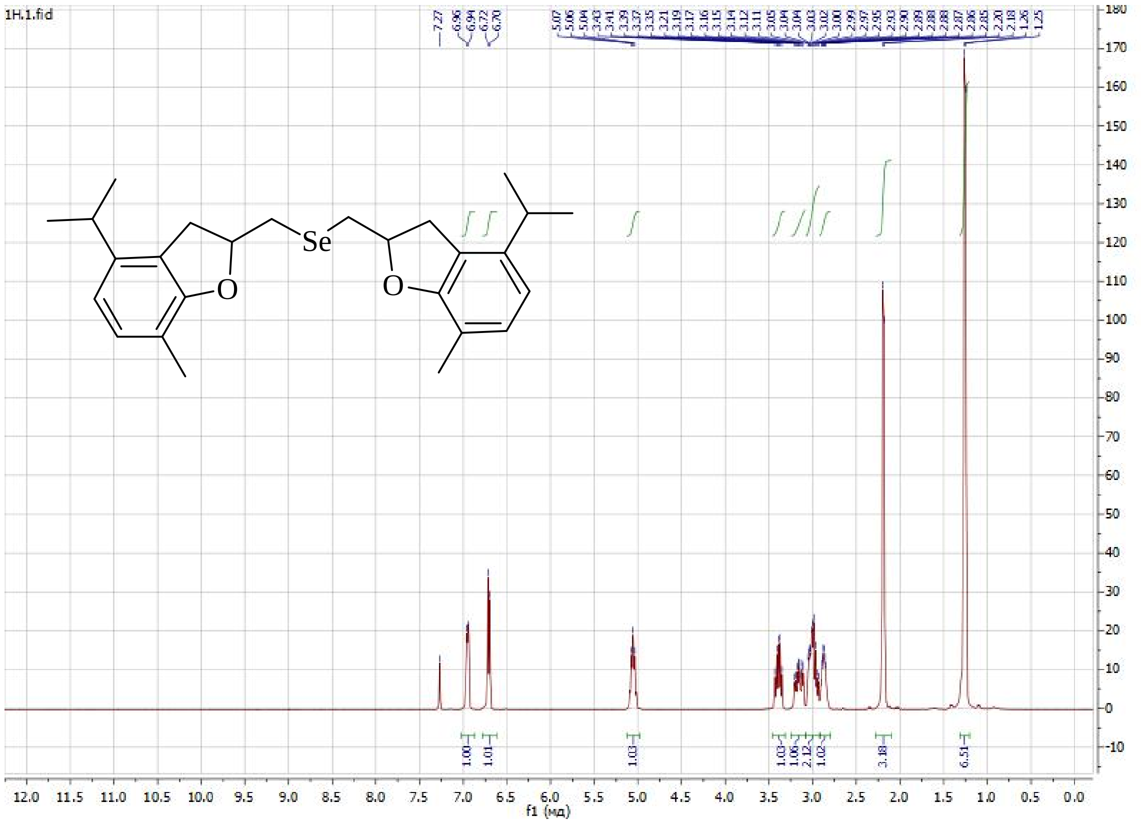

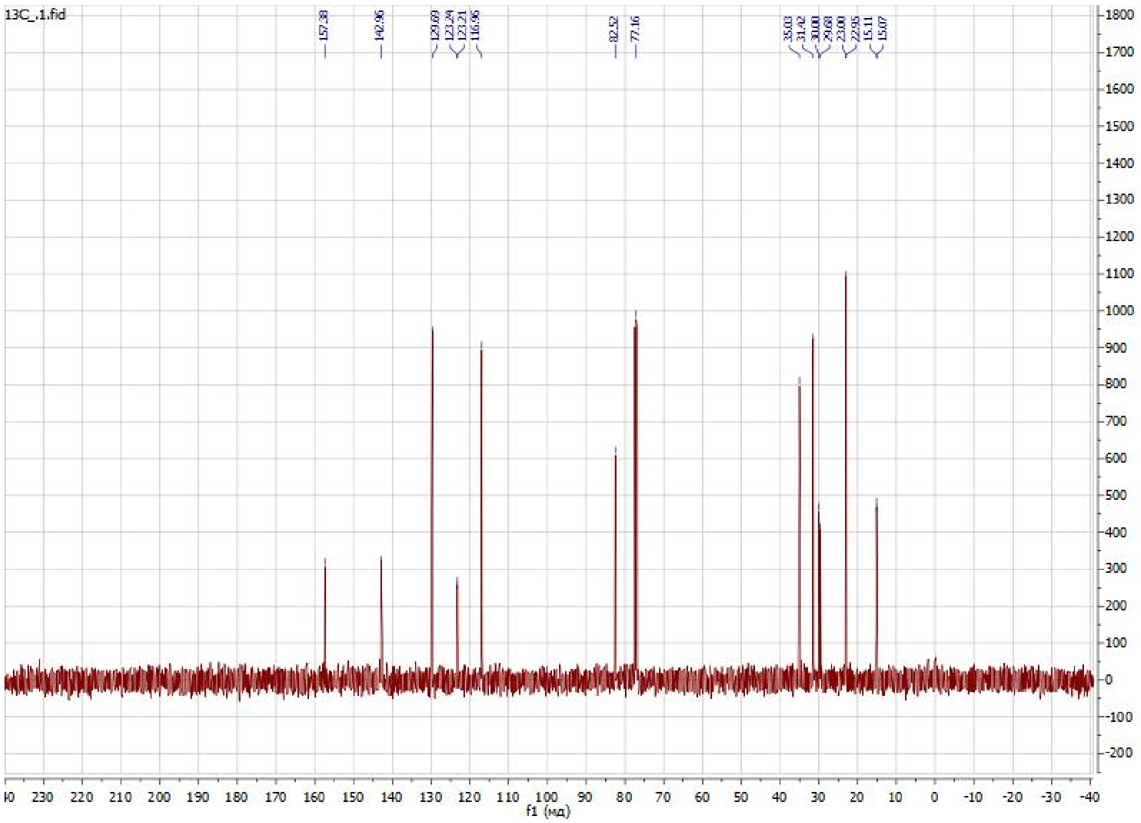

^{1}H- and ^{13}C-NMR spectra of compound 8

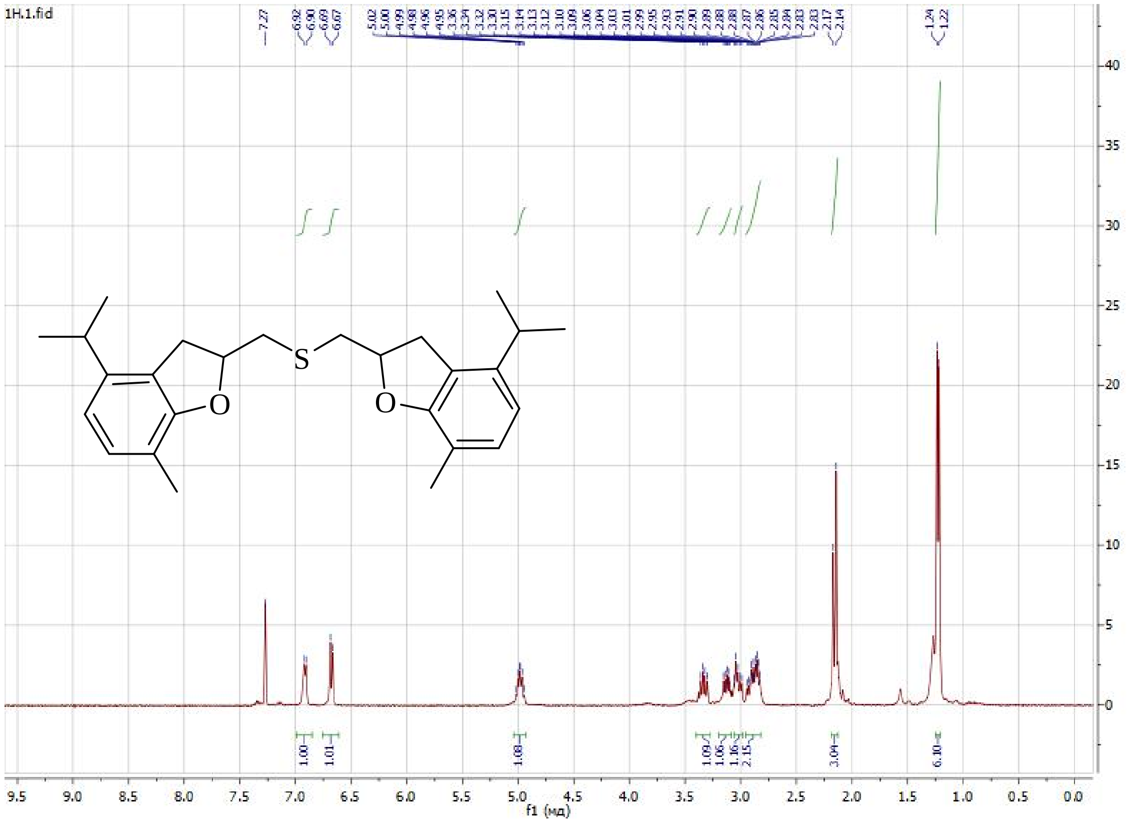

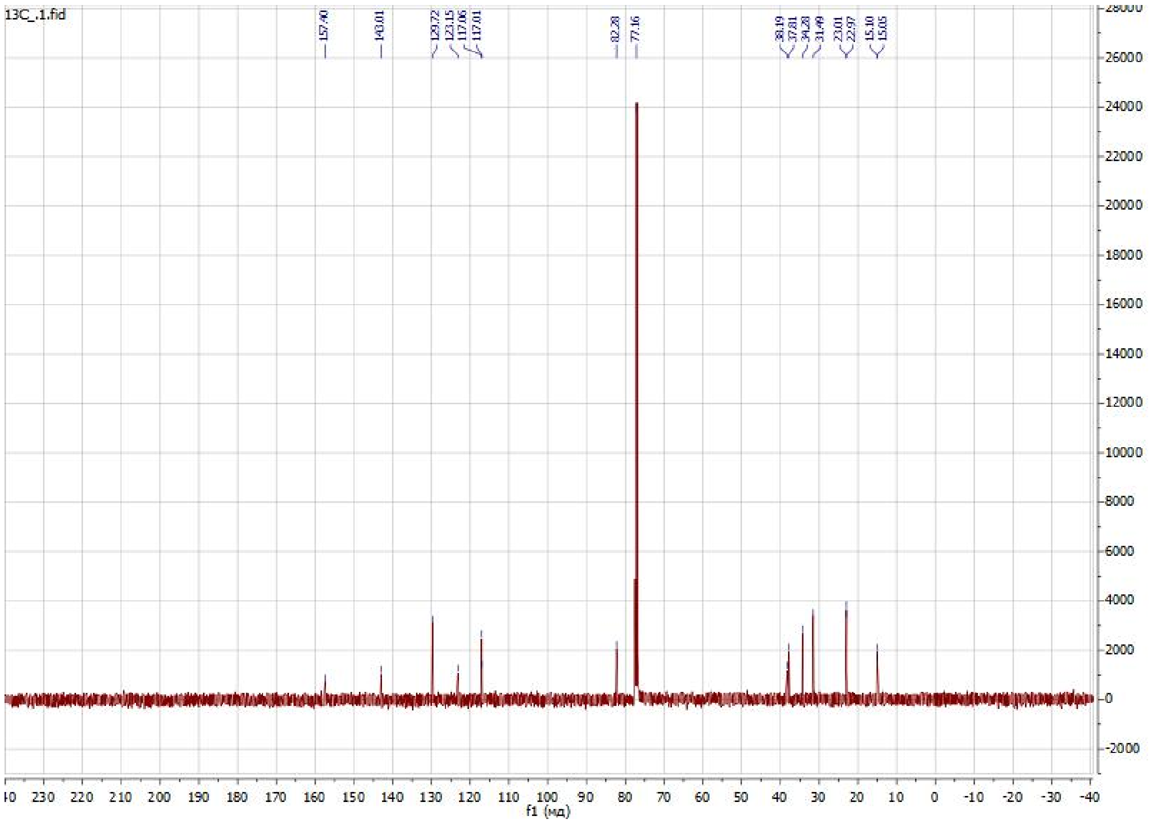

^{1}H- and ^{13}C-NMR spectra of compound 10

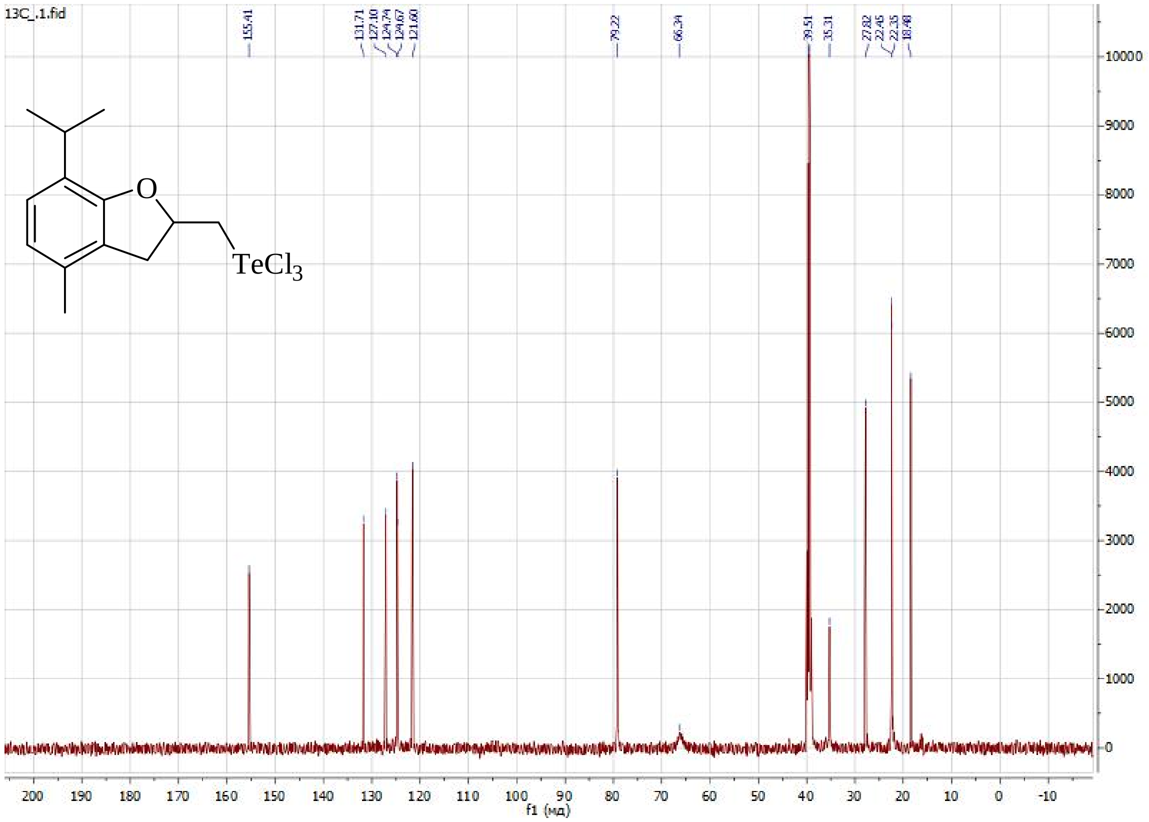

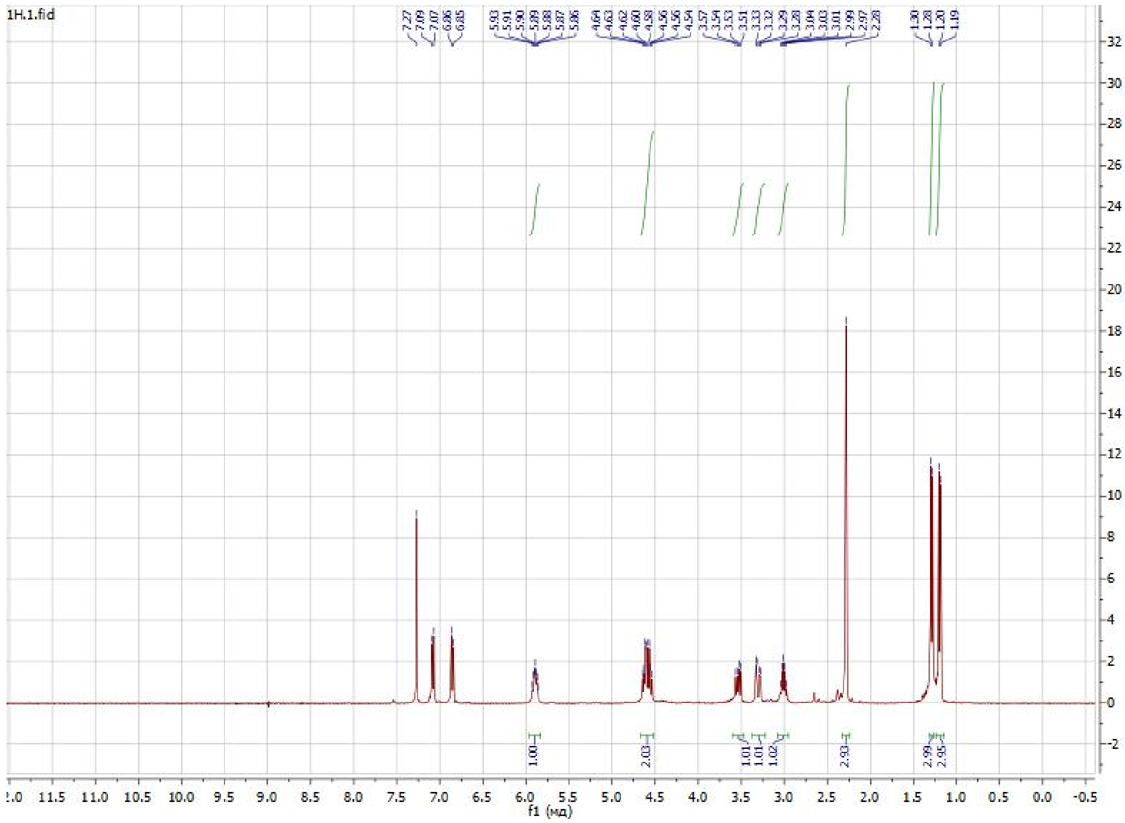

^{1}H- and ^{13}C-NMR spectra of compound 11

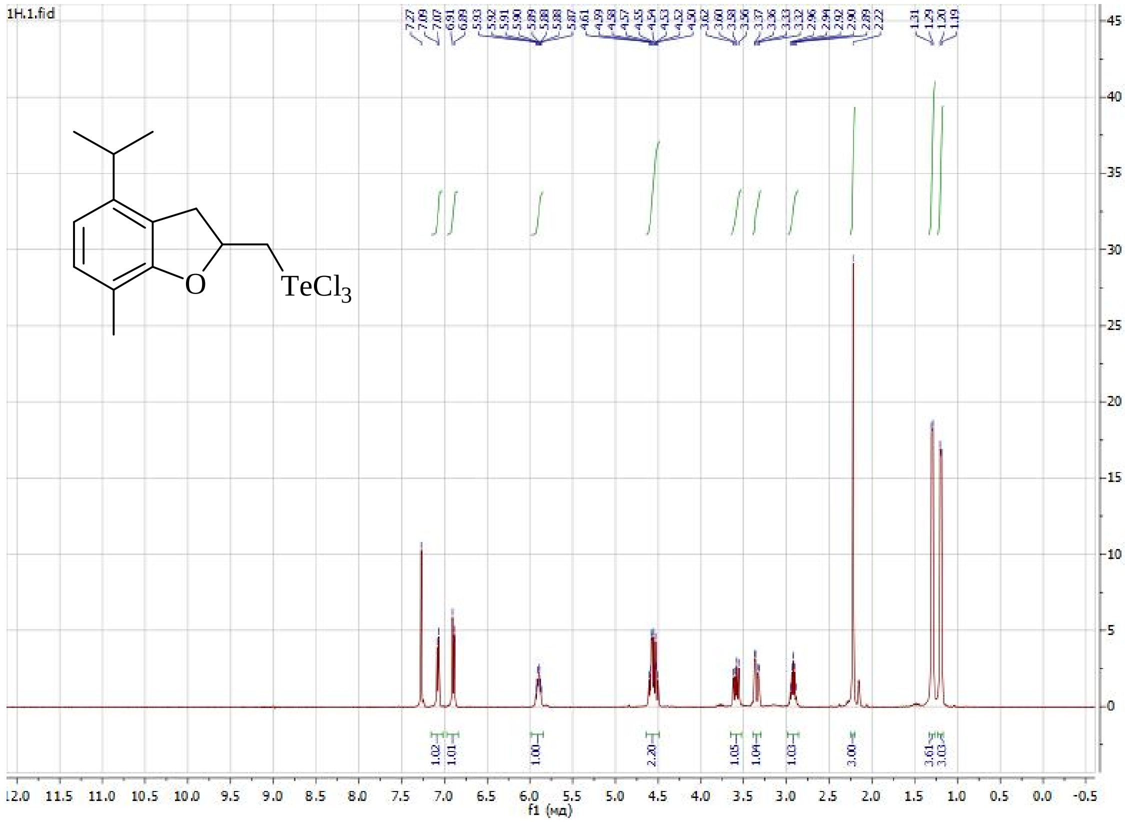

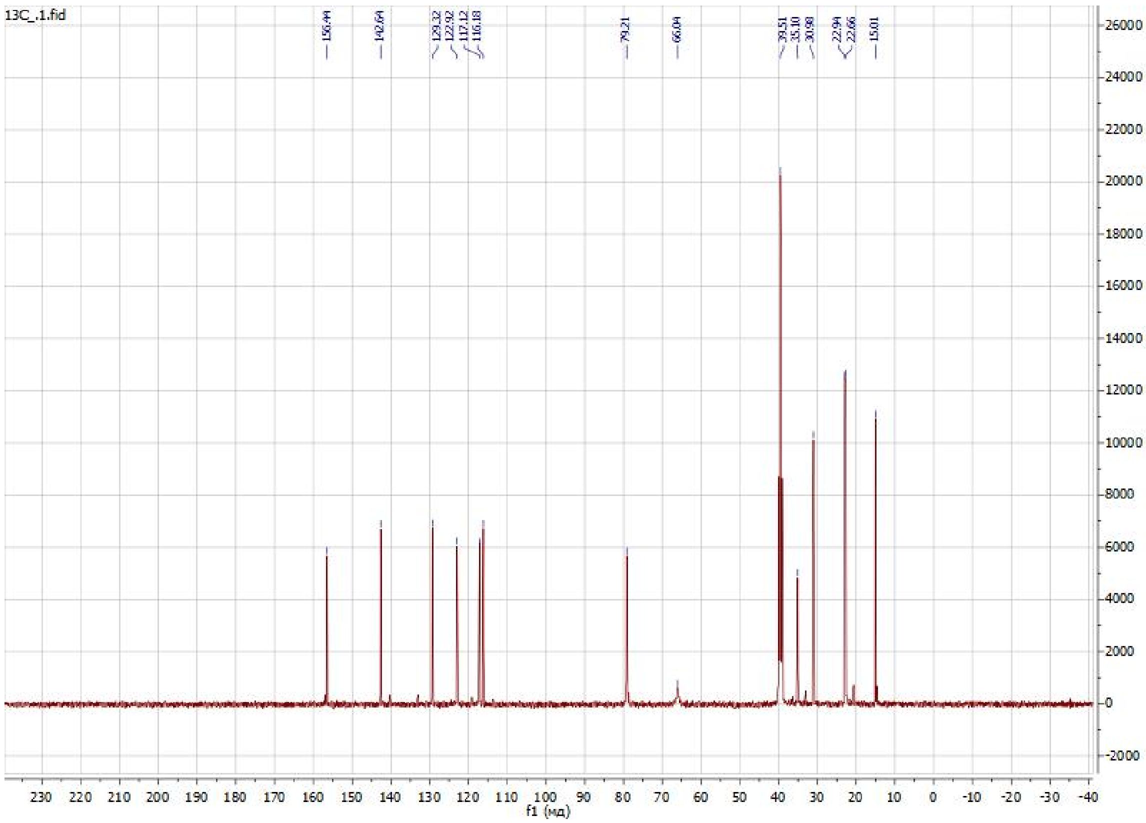

^{1}H- and ^{13}C-NMR spectra of compound 12

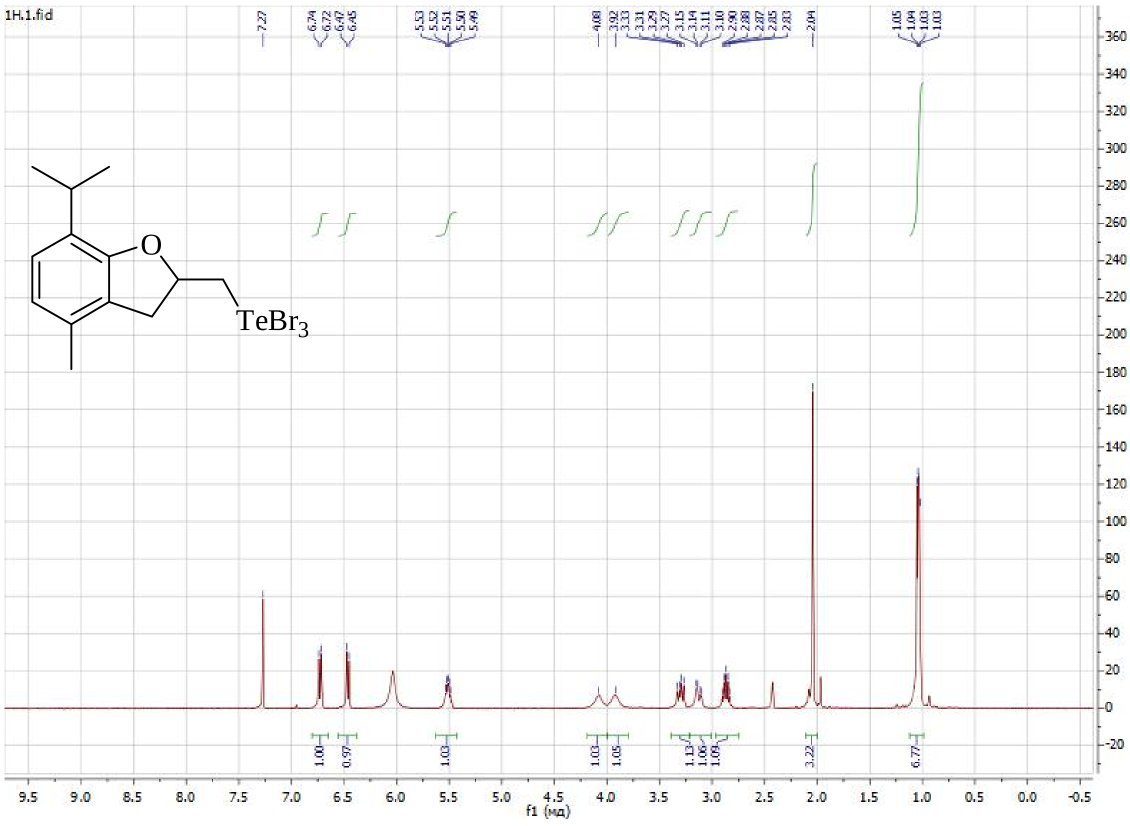

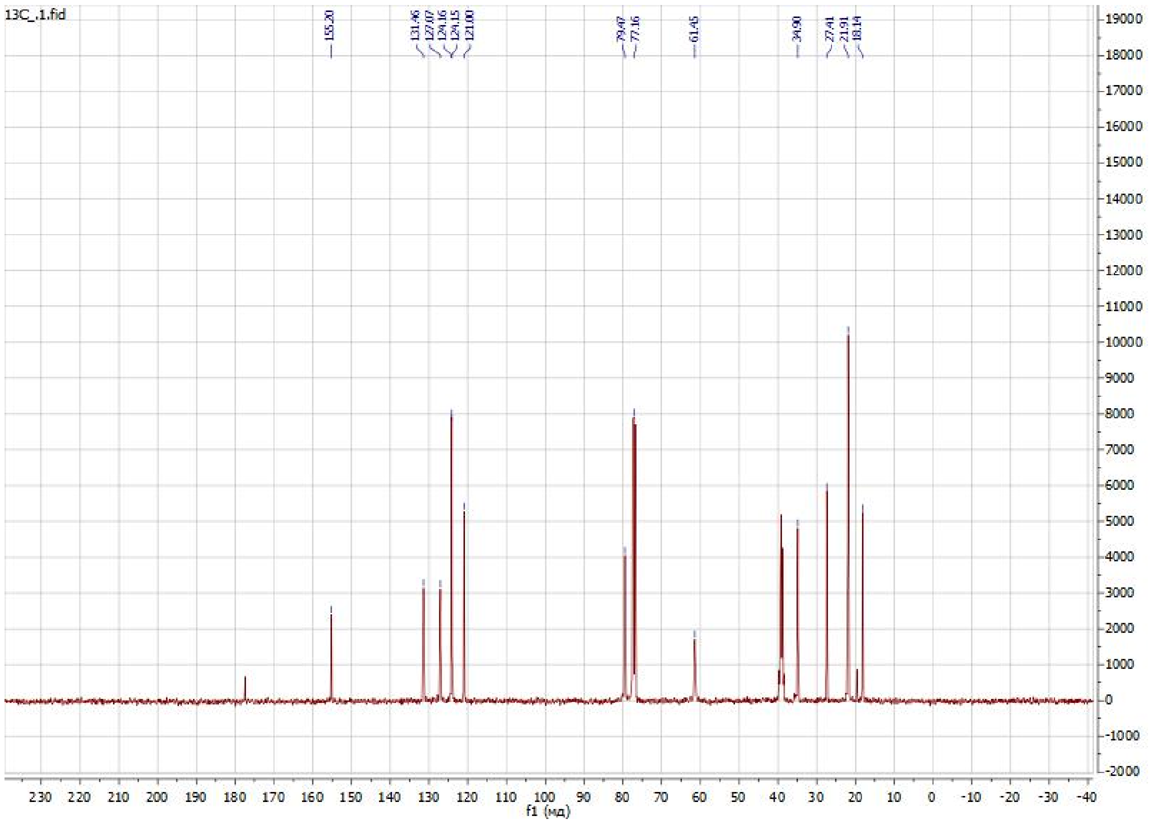

^{1}H- and ^{13}C-NMR spectra of compound 13

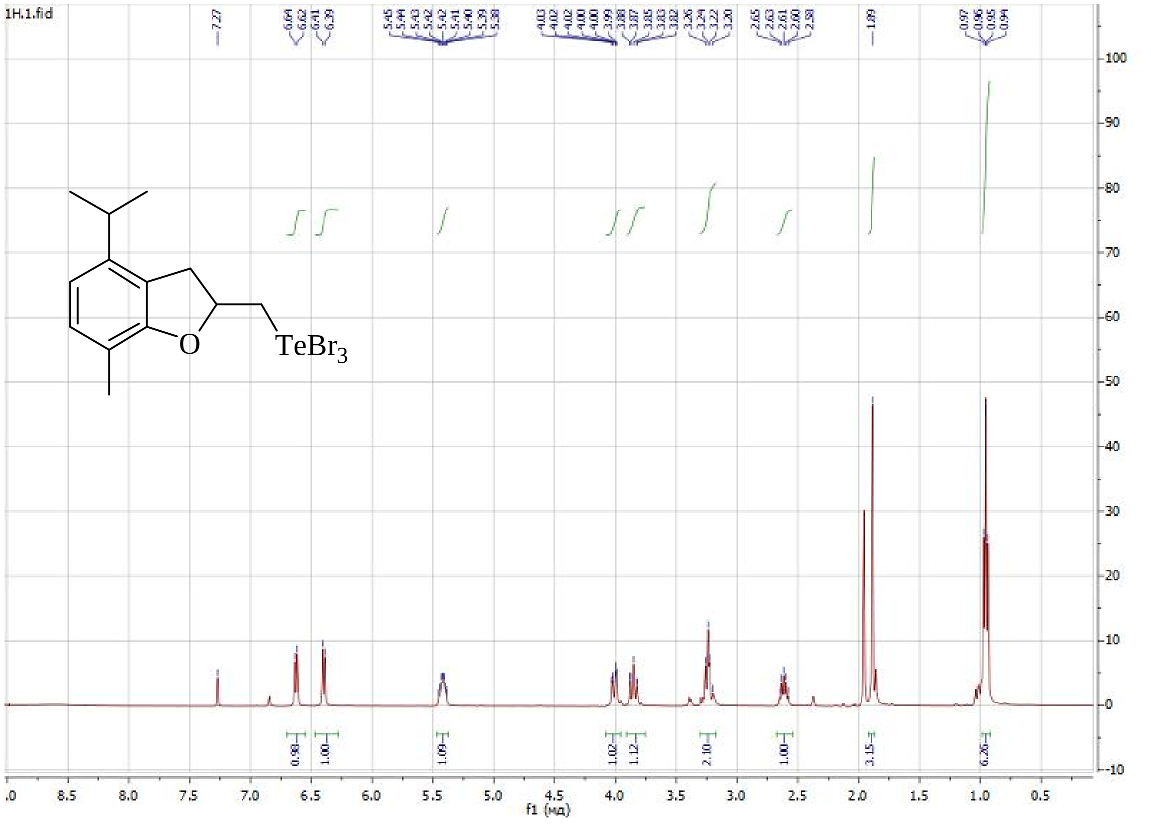

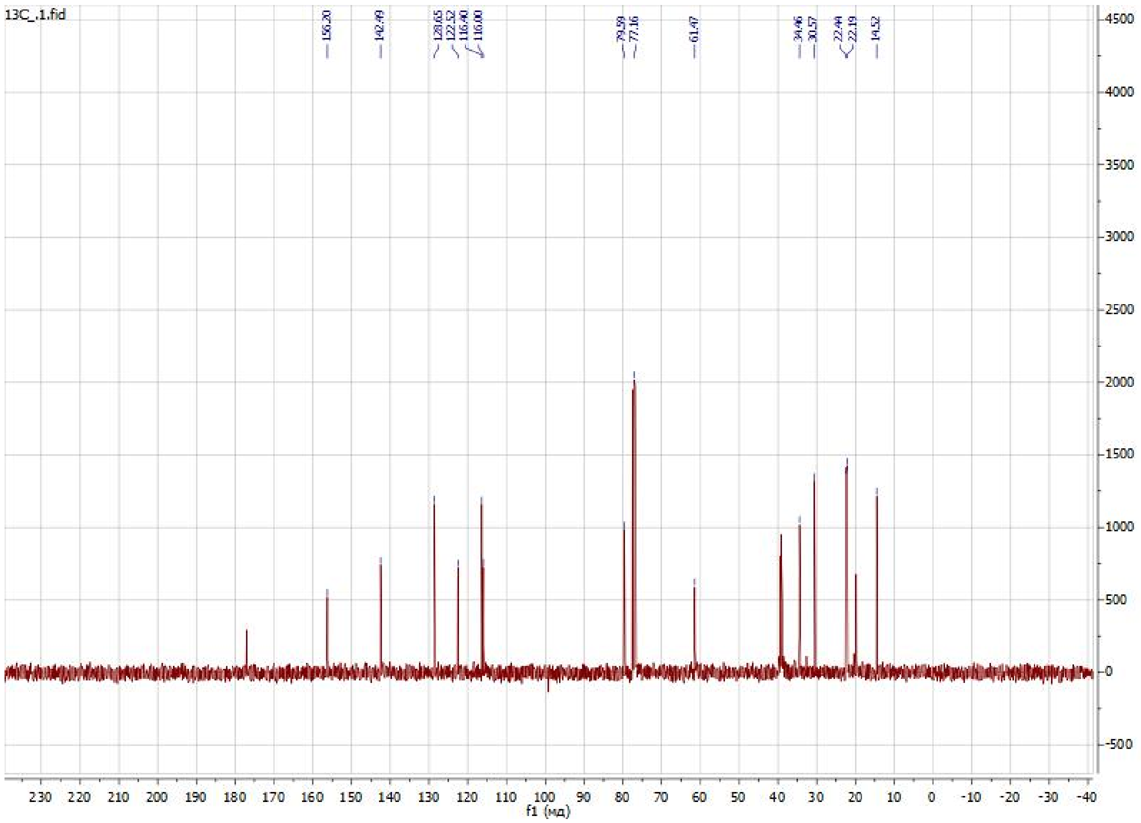

^{1}H- and ^{13}C-NMR spectra of compound 14

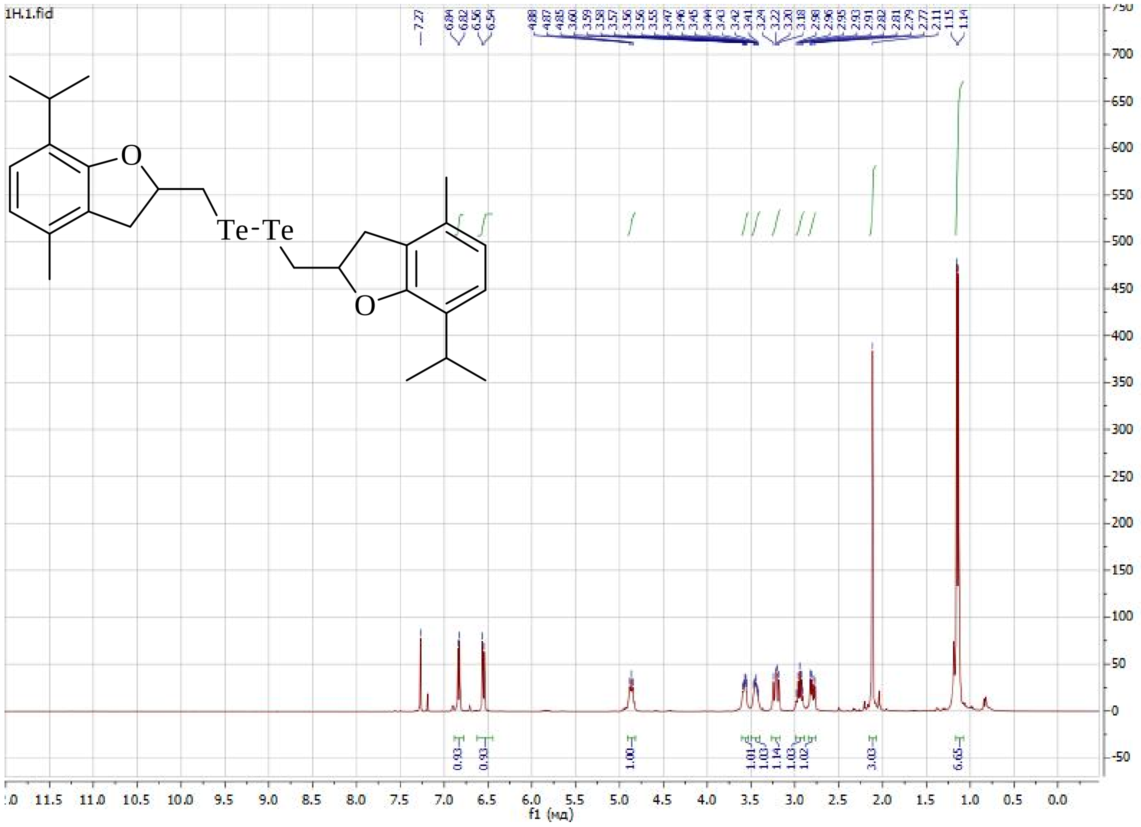

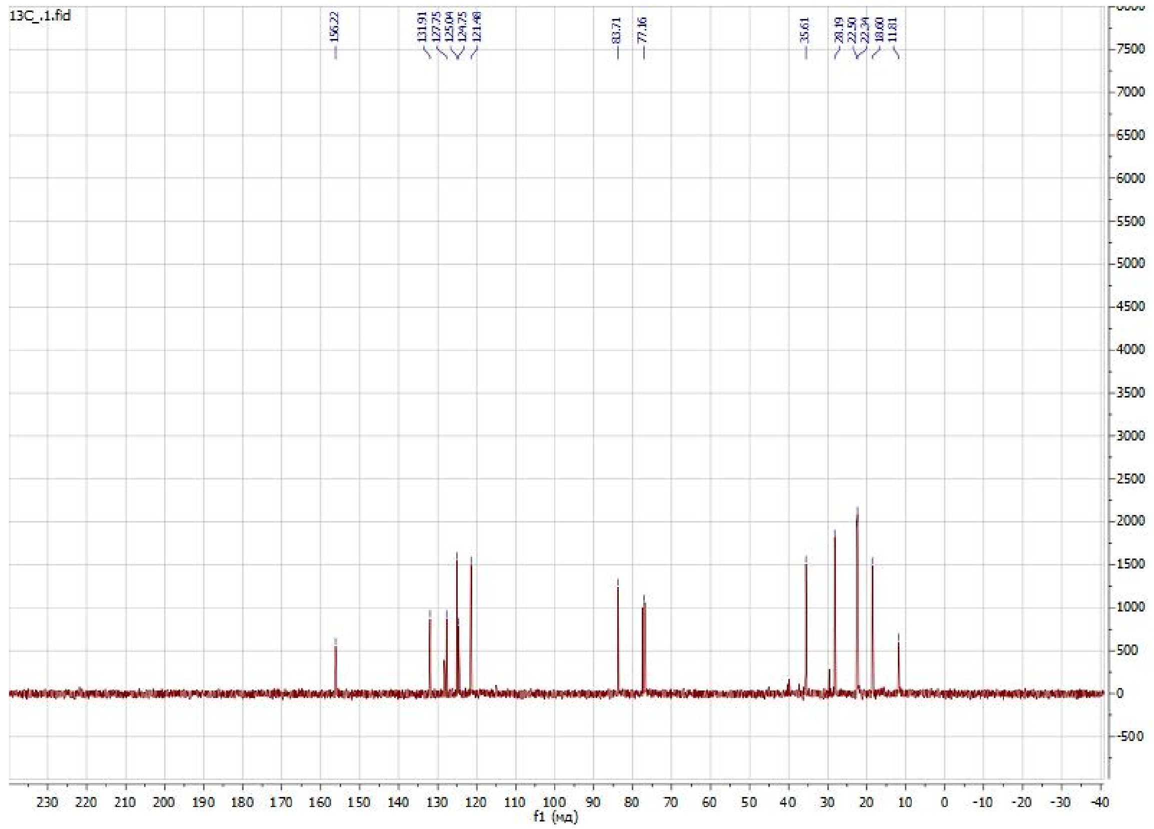

^{1}H- and ^{13}C-NMR spectra of compound 15
